# Delivering patient care during large-scale emergency situations: Lessons from military care providers

**DOI:** 10.1371/journal.pone.0248286

**Published:** 2021-03-31

**Authors:** Lara Varpio, Karlen Bader-Larsen, Meghan Hamwey, Steven Durning, Holly Meyer, Danette Cruthirds, Anthony Artino

**Affiliations:** 1 Department of Medicine, Uniformed Services University of the Health Sciences, Bethesda, Maryland, United States of America; 2 The Henry M Jackson Foundation for the Advancement of Military Medicine, Bethesda, Maryland, United States of America; 3 Graduate School of Nursing, Uniformed Services University of the Health Sciences, Bethesda, Maryland, United States of America; 4 Department of Health, Human Function, and Rehabilitation Sciences, The George Washington University School of Medicine and Health Sciences, Washington, DC, United States of America; Health Policy Research Center, ISLAMIC REPUBLIC OF IRAN

## Abstract

**Background:**

Today, physicians are at the front lines of a pandemic response. Military physicians are uniquely trained to excel in such large-scale emergency situations. Civilian physicians can harness military know-how, but it will require research into military healthcare responses—specifically, we need to learn lessons from military interprofessional healthcare teams (MIHTs).

**Methods:**

This research answers two questions: What are the characteristics of successful MIHTs? Why are those characteristics important to MIHT success in large-scale emergency situations? Using a Grounded Theory approach, 30 interviews were conducted soliciting perspectives from the broadest range of healthcare professionals who had experiences working in and leading MIHTs. Purposive sampling was used to recruit participants broadly across: contexts where MIHTs work; military branches; ranks; genders; and healthcare professions. Data were iteratively collected and analyzed.

**Results:**

30 participants were interviewed (18 male (60%); 21 officers (70%); 9 enlisted (30%)) who held various healthcare occupations (medic/tech/corpsman (9); nurse (7); physician (7); dentist (2); occupational therapist (2); chaplain (1); physician’s assistant (1); and psychiatrist (1)).

Six characteristics of successful MIHTs that are directly applicable to large-scale emergency situations were identified thatthat clustered into two themes: *own your purposes and responsibilities* (through mission focus and ethical bearing) and *get it done*, *safely* (via situational awareness, adaptability, and leadership with followership).

**Conclusions:**

This study provides insights, informed by decades of military service and training, to help civilian physicians succeed in large-scale emergency situations. These experiences from the war front can support today’s pandemic responses on the home front.

## Introduction

Until recently, physicians were not commonly expected to respond to large-scale emergency situations—largely because such circumstances rarely arose. Today, not only are physicians increasingly required to respond to active shooter incidents [1] and to domestic terrorism situations [2], but they are also on the front lines of rapidly evolving pandemic responses. All physicians need to be ready to unexpectedly engage in life-saving activities—be they caring for coronavirus (COVID-19) patients (2020, United States; 508,949 deaths and rising [3]), attending a music festival (2017, Las Vegas, NV; 58 deaths, 869 injured [4]), or dining in a café (2015, Paris, France; 130 deaths, 413 injured [5]). The fact that clinicians have selflessly responded to such large-scale emergency situations surely contributes to these statistics not being more dire. Regrettably, physicians often have little formal preparatory training for these situations because such horrors have historically not been part of civilian medical practice. In contrast, military physicians train for these circumstances because expertly responding to large-scale emergency situations is expected of all clinicians serving in the armed forces [6]. Clearly, especially given the COVID-19 pandemic, there is need for the lessons learned at the war front to be shared with the home front.

While the military healthcare system is unlike that of civilian contexts, when immediately responding to large-scale emergency situations both military and civilian physicians are thrown into very similar circumstances. At the point of injury, all clinicians work outside the traditional structures of their respective healthcare systems. Clinicians are faced with urgent casualty needs, and they need to immediately respond in situ. In these responses, military and civilian physicians must provide care without the material resources (e.g., sterile bandages), human resources (e.g., trained nurses), nor contextual resources (e.g., temperature-controlled buildings to work within) they can usually rely upon. Instead, all clinicians must make due with the resources that are at hand. Fortunately, military physicians are formally trained to engage in these scenarios [6–8]. Unfortunately, civilian physicians are *often not* formally trained to engage in these scenarios [9].

Across military contexts, patient care relies on team collaboration—military physicians almost always engage in patient care in interprofessional healthcare teams [10]. Those teams can consist of providers who know each other well, but they are often augmented with unacquainted practitioners and with service members who have minimal—or no—healthcare training. Physicians working in these military interprofessional healthcare teams (MIHTs) are expected to move nimbly across all care contexts [11]. To meet this mandate, military physicians are trained to work spontaneously, in interprofessional teams, in a diversity of contexts, with people who have a wide range of skills, with whom they have no pre-existing relationship, and to attend to patient needs that often fall outside their specialty training [6–8]. Clearly, the skills these physicians have acquired to effectively work in MIHTs in response to large-scale emergency situations could be harnessed to help civilian physicians engage in similar circumstances, including responses to COVID-19 [6].

The care delivered to patients via MIHTs during large-scale emergency situations is consistently exceptional. For example, a 10-year retrospective study of battlefield injuries sustained by the 75^th^ Ranger Regiment reported that 92% of casualties survived their injuries [12]. Notwithstanding these successes, little is known about the characteristics that successful MIHTs embody. A recent 50-year review of all the peer-reviewed and grey literature addressing MIHTs uncovered a mere 21 articles, of which only 7 reported empirical evidence [11]. This review identified characteristics supporting MIHT success (e.g., effective communication); however, the identified characteristics largely mirrored those acknowledged in the civilian interprofessional care literature. If there are characteristics of successful MIHTs that make them unique from their civilian counterparts and bolster their abilities to successfully provide patient care during large-scale emergency situations, that knowledge has not been publicly reported.

To equip all physicians to respond to large-scale emergency situations, researchers should study the MIHTs that provide patient care in these circumstances. Given the paucity of research into MIHTs, foundational exploratory studies are required. Therefore, this research asks two questions: (1) What are the characteristics of successful MIHTs? and (2) Why are those characteristics important to MIHT success in large-scale emergency situations? Using rigorous qualitative methods, the perspectives of a broad range of individuals who worked in and/or led MIHTs were solicited. By triangulating across this diversity, this investigation aims to understand what enables MIHTs to function effectively in large-scale emergency situations so that these lessons can inform civilian responses.

## Methods

The institutional review board at the Uniformed Services University of the Health Sciences approved the study. The Consolidated Criteria for Reporting Qualitative Research (COREQ) guidelines [13] informed this manuscript’s reporting.

### Participants

In this Grounded Theory study [14], purposive sampling [15] was used to recruit participants broadly across: military branches of service, healthcare professionals engaged in MIHTs, and officer and enlisted ranks (see Table 1 for full description of inclusion criteria). This maximum variation approach [15] enabled the research team to look for common patterns across a heterogeneous population. Informed consent was obtained from all participants.

**Table 1:** Demographic description of interviewees.

Phase	Number of times recommended	Gender	Branch of service	Military rank	Healthcare profession	Interview duration (in minutes)	Number of transcript pages
**1**	3	Female	Army	Enlisted	Medic	45	25
	2	Male	Navy	Officer	Dentist	75	21
	2	Female	Army	Officer	Nurse	65	22
	5	Male	Navy	Officer	Physician	52	19
	2	Male	Army	Officer	Physician	52	21
	1	Male	Army	Officer	Physician	56	28
	3	Male	Army	Officer	Nurse	75	13
	3	Male	Army	Enlisted	Medic	30	18
	1	Male	Navy	Enlisted	Corpsman	51	18
	1	Male	Air Force	Officer	Psychiatrist	80	32
**2**	2	Male	Army	Officer	Physician	22	12
	1	Male	Navy	Officer	Chaplain	41	16
	1	Male	Army	Enlisted	Medical Technician	60	21
	1	Female	Army	Enlisted	Medical Technician	39	15
	1	Male	Air Force	Officer	Nurse	58	24
	1	Male	Army	Officer	Physician	57	22
	1	Male	Navy	Officer	Physician	65	24
	1	Male	Army	Officer	Physician	28	12
	1	Male	Army	Officer	Nurse	42	15
	1	Male	Army	Enlisted	Medic	58	28
**3**	1	Female	Air Force	Officer	Dentist	47	16
	1	Female	Air Force	Officer	Physician’s Assistant	52	23
	1	Female	Navy	Enlisted	Corpsman	58	24
	1	Male	Army	Officer	Occupational Therapist	51	17
	1	Female	Army	Officer	Occupational Therapist	24	10
	1	Female	Air Force	Officer	Nurse	40	18
	1	Female	Navy	Enlisted	Medical Technician	47	16
	1	Female	Air Force	Officer	Nurse	50	17
	1	Female	Navy	Enlisted	Medical Technician	44	15
	1	Female	Air Force	Officer	Nurse	60	19
**Total**						1524	581

### Participant recruitment

The snowball technique [15] was used to find information-rich key-informants to serve as research participants. A panel of leaders in the U.S. military healthcare system were approached, requesting recommendations for service members who had served in MIHTs, led individual MIHTs, or led many MIHTs. The panel included former leaders from the Defense Health Agency, educators in the military’s healthcare training programs, and healthcare providers from each branch of service. All recommendees were contacted and asked to participate in the study. They were also asked to nominate more individuals with rich insights into MIHTs. Through this snowballing, 341 individuals were recommended from across the U.S. military healthcare system; 20 individuals received two or more recommendations. This was the study’s participant pool from which the sample was drawn.

### Procedures

A semi-structured interview protocol was developed, piloted, and revised to improve question clarity and reduce redundancy (see S1 Appendix for sample questions from the protocol). A qualitatively trained research assistant (RA), with no previous relationship with any interviewee, conducted all interviews. These one-on-one, private interviews were audio recorded, then transcribed and rendered anonymous by a third-party transcriptionist. The interviews were scheduled for one hour, but the RA allowed each participant to set the pace of the conversation and thus interview duration varied. Interviews were conducted from November 2017 to June 2018. The RA reviewed all transcripts against the original recordings to ensure accuracy.

### Data analysis

Data collection and analysis were conducted iteratively in three sequential phases. In phase 1, 10 participants who represented the broadest possible diversity across inclusion criteria were solicited, consented, and interviewed. Using Grounded Theory’s constant comparison approach [14], data were inductively analyzed using initial, open analysis. Three members of the research team (LV, KBL, MH) developed codes by reading and re-reading transcripts. Next, all research team members individually read two transcripts, then, in full group meetings, reviewed and revised the codes. Differences in understanding were resolved via discussion until consensus was achieved.

Phase 2 began by soliciting, consenting, and interviewing 10 new participants, using theoretical sampling [15] to sample broadly across the participant pool and for individuals whose insights would push the evolving analysis. Phase 2 transcripts were analyzed in constant comparison along with phase 1’s transcripts using focused, selective coding. Three researchers (LV, KBL, MH) explored which descriptive codes (in isolation or in combination) best captured the insights offered by participants, and tentatively raised these codes to conceptual core categories. The full research team then read two phase 2 transcripts. They reviewed and revised the coding structure in group meetings until consensus was reached.

In phase 3, another 10 participants, theoretically sampled for individuals whose insights would vet our analysis, were contacted, consented, and interviewed. LV, KBL, and MH analyzed these transcripts, along with data from phases 1 and 2, ensuring that all categories and codes were robustly represented (removing those that were not), modifying coding to reflect insights generated, and developing theoretical codes that organized the categories and codes into themes. The entire research team read two phase 3 transcripts, then met to inspect the theoretical coding. Consensus was achieved and the team confirmed that the study had reached theoretical saturation [13].

Analyses were confirmed in two ways. First, final analyses were shared with nine members of the panel of leaders in the U.S. military healthcare system who supported the snowball sampling. They confirmed that the analyses resonated with their MIHT experiences. Second, analyses were applied to a 2017 data set of 30.5 hours of observation data of a large-scale MIHT deployment simulation [16]. The themes and constituting characteristics identified in this study were robustly represented therein.

## Results

We interviewed 30 participants: 18 male (60%) and 12 female (40%); 21 officers (70%) and 9 (30%) enlisted service members; interview duration averaged 51 minutes, SD = 14 minutes (see Table 2 for a full demographic breakdown across inclusion criteria). The total data set consisted of 1,524 minutes of interview recordings. Six characteristics unique to MIHTs were identified (see Table 3 for the name, definition, and illustrative data excerpts for each theme) that clustered into two themes: *own your purposes and responsibilities* and *get it done*, *safely*.

**Table 2:** Demographics.

**Gender**	
Male	18
Female	12
**Branch**	
Army	15
Air Force	8
Navy	7
**Rank**	
Officer	21
Enlisted	9
**Profession**	
Medic/Tech/Corpsman_a_	9
Nurse	7
Physician	7
Dentist	2
Occupational Therapist	2
Chaplain	1
Physician’s Assistant	1
Psychiatrist	1

^**a**^A Medic, Tech or Corpsman is an enlisted service member (i.e., not an officer) with medical and healthcare training that may specialize in various patient care, treatment, or other support services [17].

**Table 3:** Characteristics unique to MIHTs.

Characteristic	Definition	Data excerpt
***Theme 1*: *Own your purposes and responsibilities***		
**Mission Focus**	Mission focus is paramount within and between MIHTs. There can be more than one mission (i.e., mission within a mission), leading to multiple mission goals (e.g., patient care, mortality). At the outset of the team—and ideally during team functioning—the mission is made clear and all team members work to achieve the mission.	“Whether or not you’re the housekeeper or the billing person or the coder, the provider, what we do here feeds into [the mission]. And everybody has to understand that. Because when you’re working in a smaller team, when you have a team that’s working with a specific patient, I think it’s very important for them to all understand what the expected outcome is for the patient and for them to all agree that they’re working towards that same outcome. If the physician is more focused on the person being able to walk and the psychiatrist is more focused on controlling PTSD symptoms, then sometimes their goals are conflicting one another and treatment plans. And I think it’s important that they come to a common understanding of establishing goals for their patient so everybody’s working towards a shared goal.”
**Ethical Bearing**	Successful MIHTs comprise of individuals who are able to work within an ethical compass. This may include situations whereby teams must make difficult decisions to care for patients or provide care for enemy combatants.	“I think on the clinical level, we’re asking health care providers to not just treat the patient; we’re asking the clinical team to assess the feasibility for continued service. We’re asking the clinical team to: ‘In your best guess, do you think that this person is going to be healed enough to return to their work on a ship or their work with the Marines or their work here?’ And we put a lot of pressure on them to make that decision. Because commanding officers of these units are counting on their people. And if they’re not going to be ready then they have to make decisions. I don’t think we put that kind of pressure on our civilian counterparts to say, ‘Hey, do you think that this guy is going to be able to–decide now if in a year from now this guy’s going to be able to go back to welding or teaching’ or whatever. I think that we rarely make them make those kinds of decisions in the same way that we do our military providers.”
***Theme 2*: *Get it done*, *safely***		
**Situational Awareness**	MIHT members have an understanding of their immediate and general environment, and they are contextually aware. All MIHT members are prepared to adjust across changing situational factors and contexts.	“Not to take anything away from them, but when you have teams that’re acclimated to being on a helicopter, that are aware of the altitude differences in the plane and how that affects respiration, that they’re going to operate in a very loud environment and they’re still going to have to have every indicator of the status of that individual available to them, whether visually or audibly, that there’s a constant vigilance shared across all those entities that see this continuum of care from being burned in a fire aboard ship to being in the burn unit down in San Antonio–they don’t necessarily know each other. But they already know they’re staging the person for that next level. So, there may be multiple more steps than you’ll have in a local injury. And so, in order to have that flexibility–cold weather, warm weather, aviation, under-sea–any combination of these variables: they impose different injuries potentially. And as a result, having an ability to pull from, if you will, on the shelf, your various specialties and capabilities, and then stage them anywhere in the world–you have to train for that. And you have to continually train for it and continually update your skills and build upon the experiences that’re historically related to you. Which is part of the legacy of every provider.”
**Adaptability**	MIHT members possess skills that allow them to be effective and operationally able to act on a moment’s notice. Importantly, successful MIHTs are made up of individuals who are able to thrive under limited resources (e.g., lack of equipment) and meet the physical demands required of them. Further, MIHT members are able to adapt to changing team composition.	“But at a certain level, you have to have the high caliber clinician who also has adaptability outside of their specific trained area, meaning I don’t … I haven’t been trained. I’m a general surgeon. But this bone is broken, and I understand carpentry enough that I can drill in an x-fix and understand kind of how it works. I’m not going to say, ‘Well, that’s not my job, so I’m not going to do it.’ So that adaptability, flexibility in the individual is critical in that environment.”
**Unencumbered Hands**	MIHT members experience a greater sense of autonomy and freedom to make care decisions. Typically, these freedoms are granted more willingly closer to the battlefield or during a humanitarian mission. This is especially common among particular roles/professions (e.g., medical technician, corpsman, medic). This includes references to the scope of practice that military healthcare team members can have that are not the same as in civilian contexts.	“… from my standpoint and my experience and career has been there are special operations combat medics in the Army. Between them and our Green Beret medics, the 18-Deltas … there is no equivalent to them in the civilian world … … they need to be able to shoot really well, and jump out of airplanes, and talk foreign languages and all this; but we also expect them to be able to do the primary care stuff of taking care of that team when they’re sitting on a base for two, three months and they’re really just worried about diarrheal disease from not cleaning the kitchen right, or sanitizing the water so you can brush your teeth, to, ‘Whatever that animal looks like, it’s got some zoonotic disease, so stay away from it,’ et cetera. So, they probably are the most unique aspect of our healthcare teams, and I mean there is no equivalent… …[we’re] taking our non-licensed healthcare providers, our medics and teaching them to do life-saving skills on the spot… …we’re trying to practice world-class medicine in less-than-world-class situations…”
**Leadership & Followership**	Leadership: A team leader who is or group of individuals who are able to take on leadership roles within the team. Ability to act respectfully, encourage team members, identify needs of the team and the patient, work adaptively, and understand both team- and self-weaknesses. A strong leader will not need to be present at all times for a team to be successful. Followership: Successful MIHTs are composed of good followers who are able to act supportively and anticipate the needs of other team members, the leader, and the broader mission.	“I think sometimes people think when we hear ‘followership’ it’s like a herd of sheep just following along, but I don’t think that’s a really great explanation. There’s always, you know, you need a leader, and we can’t have a bunch of leaders, you know, too many chefs in the kitchen is gonna cause just problems and errors and mass chaos, and so a little bit of knowing your role and when we have a defined leader, being able to follow and do what needs to be done in that situation. And still towing the line and holding standards and stuff like that.” “But I think [there’s] an aspect of respect that’s important too, and respect working both ways, from the leader to the follower and follower to the leader.”

### Theme 1: Own your purposes and responsibilities

MIHT collaborators bear the weight of the goals and obligations of delivering patient care in challenging situations. To meet that charge, team members draw on mission focus and ethical bearing.

#### Mission focus

When MIHTs engage in patient care, there is an overarching objective—a mission—that informs and directs the team’s efforts. At the outset of any engagement, the MIHT’s mission is explicitly described thereby ensuring that each collaborator can focus their efforts towards the realization of this aim. As one participant explained: “it’s important that all the teams have an understanding that the overall command mission is to support war-fighter readiness and taking care of their families. Everything we do is to support that.” The mission becomes an orienting objective for all subsequent decisions. Mission focus helps MIHT collaborators harmonize their efforts towards a common goal.

#### Ethical bearing

MIHTs often confront moral dilemmas when delivering patient care, especially in large-scale emergency responses. To navigate these challenges, MIHTs rely on a shared ethical bearing. This common moral compass helps clinicians decide which patients will receive the limited resources at hand. One participant described a situation where, in combat, two patients were rushed into the care facility: one soldier wounded by an improvised explosive device and one enemy combatant who set the device. The entire MIHT agreed that “it was the right thing to try and save their [enemy combatant’s] life” as well as the soldier’s life, despite the fact that surgical resources were in limited supply. MIHTs regularly face such dilemmas and need to embody clear ethics and moral strength.

### Theme 2: Get it done, safely

When delivering patient care, the MIHT collaborators exercise characteristics that enable them to be nimble. Physical safety, material resources, skilled collaborators—none of these are guaranteed. To succeed despite these unstable foundations, MIHTs harness four specific skills: situational awareness, adaptability, unencumbered hands, and followership with leadership.

#### Situational awareness

For MIHTs, situational awareness entails two things not typically required of civilian teams. First, MIHTs must be aware of the physical and human resources available. MIHTs often work in contexts with limited supplies requiring collaborators to be strategic with how they use those resources. Second, situational awareness requires being attentive to the physical environment and threats present therein. In combat, situational awareness demands being cognizant of enemy combatants and potential physical threats. In humanitarian deployments, that vigilance typically focuses on geographical instabilities, on pathogen contamination, and similar threats. As one participant explains, a MIHT member must have his/her “head on a swivel” to stay safe. When MIHTs offer care, team members must continually read the environment to understand the physical situation and the resources available therein, so that they can adjust their actions straightaway.

#### Adaptability

Adaptability enables clinicians to be effective care providers at any given moment—regardless of the resources at hand or the changing environment. During deployments, team members strive to keep themselves and their patients safe. This can require building improvised shelters or using medical equipment in unexpected ways (e.g., making tourniquets out of IV tubing). Military clinicians also need to be physically and psychologically able to meet a variety of demands—be it calming a hysterical patient or providing care in suboptimal contexts (e.g., at the point of injury or in makeshift tents erected to house patient overflow). They also need to be effective clinicians when resources are limited or unavailable; as a result, MIHT collaborators rely on adaptability to construct solutions for patient care needs when few or no medical supplies are at hand. Finally, MIHT collaborators will often be required to administer patient care that is not niched within their sub-specialty. As one participant explained, in his normal work as a general surgeon, he would not be called up on to care for a patient with a broken leg because an orthopedic surgeon would be brought into the team. But, as a MIHT collaborator working in contexts where no other surgeon is available, he needs to be ready to provide this care. As he explained, MIHTs need versatile clinicians: “you have to have the high caliber clinician who also has adaptability outside of their specific trained area.” Adaptability requires MIHT collaborators to be physically and mentally capable; it necessitates being flexible, resourceful, and clinically prepared to provide patient care in sub-optimal environments.

#### Unencumbered hands

MIHT clinicians are often accorded greater autonomy and freedom than their civilian counterparts when working in deployed contexts. MIHTs regularly require every team member to use every aspect of his/her skill set. When serving in humanitarian or combat contexts, MIHTs cannot restrain team members with concerns about professional scope of practice regulations or interprofessional turf wars. As one participant explained, members of the MIHT are expected, “at a moment’s notice, [to] be able to manage multiple complex trauma patients at the point of injury while there’s still shooting and blowing up going on.” There is no time to be hampered by policies stating that a medic is not qualified to clamp an artery. If the medic has the skill and if the skill is needed, the medic will be asked to use that skill. To be successful, the MIHT empowers every collaborator to bring the entirety of his/her skill sets to the patient care activity. With their hands unencumbered by policy or political restraints, the MIHT has a broader skill base to draw upon.

#### Leadership with followership

Members of MIHTs recognize the contribution of the leader to a team’s success. An effective MIHT leader will: inform all MIHT members of the mission; model moral clarity; ensure the team’s safety; and support adaptability. Members of the MIHT assist leaders to meet these mandates through followership—i.e., the willingness to actively support leaders. Followership activities that MIHT collaborators mobilize include: anticipating the team’s needs and preparing for them; offering advice to the team leader; and recognizing when the team is missing a skill and offering to fill that gap. Followership is a critical skill required of every MIHT member. As one participant explained: “because of your role, you [the physician] end up a lot of times being the team leader, but part of being a leader is also being a follower and being smart enough to know that the nurse knows what he’s doing.” As situations dynamically evolve, all MIHT members are prepared to be both leaders and followers.

## Discussion

The sharing of lessons learned on the battlefield with homeland hospitals has historically greatly benefited civilian trauma care (e.g., the merits of tourniquets for hemorrhage control were proven during the wars in Iraq and Afghanistan and have been reported as significantly improving the outcomes of the Boston Marathon bombings [18]). In 2016, the National Academies of Sciences, Engineering, and Medicine published findings from a committee’s investigation into how continued improvements in military and civilian trauma could be secured by ensuring that lessons learned on the battlefront were shared, sustained, and built upon in civilian healthcare. This report highlighted the lethal paradox separating military and civilian sector trauma care:

“On one hand, the nation has never seen better systems of care for those wounded on the battlefield or severely injured within the United States. On the other hand, many trauma patients, depending on when or where they are injured, do not receive the benefits of those gains. Far too many needlessly die or sustain lifelong disabilities as a result”

[6].

The report offers 11 recommendations to improve trauma care, several of which note the importance of trauma *teams* to successful patient care. However, to date, researchers have yet to investigate the characteristics that enable MIHTs to effectively engage in immediate responses to large-scale emergency situations [11]. This study begins to fill that gap. This investigation found six characteristics supporting successful MIHTs that can be organized into two themes: (1) *own your purpose and responsibilities*, and (2) *get it done*, *safely*. We acknowledge that research into civilian interprofessional healthcare teams have noted the existence of some of these characteristics (e.g., leadership with followership [19–22]). However, this investigation highlights how these six characteristics are uniquely combined and relied upon by MIHTs to provide high-quality patient care under the most challenging circumstances.

These two themes, and each characteristic embedded therein, could help civilian physicians respond to large-scale emergency situations such as mass shootings or contending with an overflow of COVID-19 patients. First, when physicians find themselves in the midst of a large-scale emergency situation, our data suggest that they should first identify and accept their role, purpose, and responsibilities. This means knowing the mission (e.g., to attend to as many patients as possible while remaining uninfected themselves) and working towards the achievement of that mission with ethical clarity (e.g., allocating ventilators via explicitly defined moral considerations). By making team members aware of the mission and by modeling ethical behavior, physicians can help to ensure that the team’s efforts are aligned toward fulfilling their goals.

Second, our data suggest that physicians should focus on safely achieving their goals. This requires that physicians be situationally aware of the physical environment, the threats therein, the resources available, and the evolving team activities. With this awareness, physicians can meet patient needs with adaptability by being physically and mentally resourceful (e.g., using social media to request donations of personal protective equipment [#GetMePPE] [23]). The physician must learn to see each person as having a diversity of skills that can be put to work to offer patient care—a perspective that can be very difficult given the intensity, complexity, and challenges of the situation. The physician must also be prepared to have team members harness the full extent of their clinical skill set when providing care, without limiting this engagement due to concerns about the professional boundaries. Finally, our data suggests that physicians are both leaders and followers. Members of the team might have contextual information that the physician needs to know (e.g., status of COVID-19 patients in other hospital wings) to keep the team and patients safe, or they might have skills and/or resources that the physician can use. In these high-stress care contexts, exploiting the expertise and insights from all team members will support the accomplishment of the team’s mission.

## Limitations

This study has strengths and limitations. This research is the first to describe MIHT characteristics of success that can be used by civilian clinicians in large-scale emergency situations. Although the study is strengthened by the diversity of the participants, the analysis is based on interview data from 30 military healthcare providers. Moreover, the data are from volunteer participants; selection bias likely exists. Despite these limitations, the study generated insights that can help civilian clinicians respond to large-scale emergency situations. Future work should collect additional data from a more representative sample of MIHT collaborators. Our own forthcoming work aims to do this by surveying a larger, diverse sample of healthcare providers from across the U.S. military health system. Such broader scope data collection can use the findings from this study to then explore which characteristics are most important to patient care activities during specific times in large-scale emergency situations.

## Conclusions

Several factors contribute to the success of interprofessional healthcare teams in military healthcare contexts. Six characteristics of MIHTs, that can be arranged into two themes, are actionable by civilians engaged in large-scale emergency situations. They are: (1) own your purposes and responsibilities by having a mission focus and an ethical bearing; and (2) get it done, safely, by maintaining situational awareness, being adaptable, and functioning as strong leaders and followers. By harnessing the lessons learned from the military, civilian physicians may be better equipped to respond to large-scale emergencies, such as the COVID-19 pandemic.

## Supporting information

S1 Appendix (DOCX)

S1 Dataset (PDF)
