# Supplementary material for: Delivering patient care during large-scale emergency situations: Lessons from military care providers
Source: PLoS One. 2021 Mar 31;16(3):e0248286. doi: 10.1371/journal.pone.0248286 (PMC8011761; doi:10.1371/journal.pone.0248286)
Supplement: S1 Appendix — (DOCX) [file pone.0248286.s001.docx]

**Appendix A: Interview Protocol**

| ***Question Set #1: What are the characteristics of a successful military interprofessional healthcare team?***  One of the major objectives of our study is to describe the characteristics of a successful military interprofessional healthcare team. To that end, we’d like to ask you about what a successful team means for you.  ****NOTE: During question set 1, it is* ***imperative*** *that the* ***interviewer keep track of the characteristics*** *that the participant identifies as unique to military care teams. This list is needed to start question set #2.* |
| --- |
| 1. What are the characteristics of a successful military interprofessional healthcare team?   *If respondent requires a re-phrasing of the question:*  If you think of the different interprofessional healthcare teams that you’ve seen in the military, what made some team particularly effective in their work?  As a follow up to that question, the definition of success that you just provided – is that specific to teams in a specific context or is it broadly applicable to military interprofessional care teams in any context?  *If their definition is not transferable to other contexts*: Why is it specific to ‘x’ context? *(x=name the context they described)*  *If it is transferable:* Can you explain why that definition of success is relevant to all military healthcare teams? |
| 1. I’d like to ask you to think back across the care teams that you either participated in as a healthcare professional and/or the teams you oversaw as a leader. Can you tell me a story of an event when a military interprofessional healthcare team proved themselves to be particularly successful?   “That’s an interesting story. As you think on that situation, what are some of the things the team did or the qualities they displayed that enabled them to be successful? If you had to label that situation with qualities that enabled success, what labels would you create?” |
| 1. Can you tell me what makes interprofessional healthcare teams that work in the military different from those that work in civilian settings?   *If the participant needs a re-phrasing of the question:* If you think back on your experiences, what distinguished healthcare teams that worked in military contexts (such as combat deployment, or humanitarian contexts, or at centers like Walter Reed) from interprofessional teams that work in civilian settings (such as the in-patient settings at civilian tertiary care hospital)? |
| ***Question Set #2: Why are these characteristics important***  Now that we’ve had a chance to discuss the differences between civilian and military healthcare teams, and we’ve discussed the characteristics of what makes military care teams unique, I’d like to now ask you to reflect on why those differences exist. |
| 1. During our conversation thus far, you’ve noted the following characteristics… that military interprofessional healthcare teams are different from civilian teams because ______________ *(list items identified in question set 1).*   Refer to list. Am I right when I summarize as x, y, z?  Is that summary correct? Did I miss anything?  With that list in mind, can you tell me why those characteristics are important for military interprofessional healthcare teams? Why are the characteristics you’ve listed as “successful” important for military interprofessional healthcare teams?  *If the respondent requires a re-phrasing of the question:*  What are the things that those characteristics enable the team to do or to be that aren’t things that civilian teams need? |
| 1. Next, again thinking back on all of your experiences, can you explain to me why the US military needs interprofessional healthcare teams that are specifically trained to work in the military?   Can you tell me more about that? Why x y z? |
| ***Question set #3: Reflecting on the participant’s healthcare profession background, what is a successful military _____***  ****** Skip this question set if the participant is not trained as a health professional******  In this question set, we’d like to know your thoughts as a health professional who works/worked in military healthcare teams. |
| 1. I’d like you to think back on all your experiences in healthcare. Can you tell me what makes a military _______ different from a civilian ______? **insert name of profession identified in demographic question 2*   Why is that difference important?   1. Based on your experience, how do you define a successful military interprofessional healthcare team? |
