# Supplementary material for: Delivering patient care during large-scale emergency situations: Lessons from military care providers
Source: PLoS One. 2021 Mar 31;16(3):e0248286. doi: 10.1371/journal.pone.0248286 (PMC8011761; doi:10.1371/journal.pone.0248286)
Supplement: S1 Dataset — (PDF) [file pone.0248286.s002.pdf]

Military Interprofessional Healthcare Teams Study

**This data has been collected from active military members involved in active deployments. Therefore, in our minimal data set, we have had to redact the transcripts for: 1) participant anonymity; 2) sensitive military information; 3) any and all references to locations, years, or size of deployment body.*

Interview A

[Redacted to protect participant anonymity]

Interviewer: Okay. So one of the major objectives of our study is to describe the characteristics of a successful military interprofessional health care team, so to that end, we'd like to ask you about what a successful team means for you. So what do you think the characteristics are of a successful military interprofessional health care team?

Interviewee: So I think the most critical thing – right off the bat - is individual currency and competency.

Interviewer: Can you expand on that?

Interviewee: So every member of that team, in their defined specialty – medic, surgeon, nurse, anesthesia provider, doesn't matter – needs to be current and competent. So, current means up to date with the latest techniques and procedures; competent means able to perform to a measured level of proficiency. If every member of the team is not current and competent, the team fails, especially in these high-risk environments. So for example, as a *[healthcare profession]*, I'll go into *[hospital]* in the middle of the night and essentially assemble a group of mostly strangers, but they all bring currency and competency to the table and we're able to deliver.

Interviewer: How do you think you could implement successful teamwork if one of the team members is not current or competent?

Interviewee: It becomes – there are obviously situations where that happens, and you're obviously able to overcome that, but it comes with real costs, costs in time to care, risk of errors, and the like. So, optimally, you need to avoid that as a – that needs to be your basis for any teamwork.

Interviewer: Right. So are there any other characteristics you can –

Interviewee: Sure. So the next thing is, folks need to be aware of the environment which they're in. It's more critically important the more austere the environment becomes. And it's the physical environment of where you're working, but it's also the larger construct of the system which you're working in. When you're at a hospital like *[hospital]* and you're working a location like that all the time, the environment's very familiar and the system is well optimized. When you're in a far forward environment, the environment becomes more austere. There's limitation on the resources. And you need to be aware of the larger battlespace and your role in that battlespace. So awareness of the environment and then the roles of the other individuals is key.

Interviewer: So how do you think that a – for a team to be successful, adapting to, like you said, it's more critical in a more austere environment. What characteristics do you think that they have to bring to the table that might be different from being here in garrison?

Interviewee: So they need to be trained to their baseline currency and competency, but not just for routine care but for what they need is in that expeditionary environment, as a basis. And then if we're talking about these austere teams, they probably need some level of just-in-time team training. And that's familiarization with the environment that they're going into, situational awareness of that environment, and what their individual roles are. 'Cause oftentimes in those deployed environments, folks may fill more than one role. That's not always as clear as it is in a standard hospital.

Interviewer: Okay. So I'd like to ask you to think back across the care teams that you've either participated in as a health care professional and/or the teams you oversaw as a leader. Can you tell me a story of an event when a military interprofessional health care team proved themselves to be particularly successful?

Interviewee: Sure, so our experience in *[deployment location]* is a great example of that. *[Deployment location]* was a role 3 hospital… that was a *[redacted to protect participant anonymity]*. So, large mix of health care providers, from not only different specialties and disciplines but also different cultures.

Interviewer: Interesting.

Interviewee: So it actually was a great study in team dynamics.

Critical to that success – and it was wildly successful. During a period of *[timeframe]*, we had…– we took care of *[number]*

trauma patients. We did *[number]* operations on those *[number]* trauma patients. We gave *[number]* units of blood. These were patients at the highest level of injury severity, and the died-of-wounds rate was about 3 percent. So if you showed up with vital signs, you lived.

And the pace was very intense. It was a marathon, not a sprint. But everyone was able to get through that cycle, and there was a significant amount of turnover, kind of, even during our *[timeframe]* rotation of some of the individuals. But with all of that, we were able to deliver the mission, and folks left with a lot of professional satisfaction, which was not always the case in those settings.

And key – and it boils down to essentially three things, I think: having a well-defined mission – and in that environment it's really obvious the mission is taking care of the blown-up guy or gal; having the right people – that goes back to the currency and competency, and oftentimes in those settings, you are sent the people. You need to make sure that they very quickly assess whether they are well matched to what their roles are intended to be. And the third piece is effective communication. And that's critical and often – look, and that's not just having meetings. It's effectively communicating.

Interviewer: So when you say "not just having meetings," what do you mean by effectively communicating?

Interviewee: It's multifactorial. Yes, absolutely, you need meetings. You need clinical care meetings where you get the group together and talk about the care. You need multidisciplinary rounds. There's one-on-one meetings. It's the full gamut of communication.

Interviewer: So can you tell me what makes an interprofessional care team that works in the military different from those that work in civilian settings?

Interviewee: The mission. And the closer you are to the mission, the more focused you are and the more extraordinary the results are.

Interviewer: So what do you think – how does the mission compare from the military perspective versus the civilian perspective?

Interviewee: So the mission of the military is to take care of the blown-up guy or gal, take care of our nation's heroes. The mission on the outside is oftentimes, ideally, is to provide the best, highest-quality care

possible to patients. But that mission becomes diffuse when passed through, oftentimes, the filter of reimbursement, bureaucracies, everyday lives, which is no different than what you face at a stateside military treatment facility. The mission in *[deployment location]* or *[deployment location]* or on a ship or far-forward becomes very focused. As you step back through the continuum of care, back towards stateside facilities, it becomes less focused. And depends on how many – the flow and the influx of the casualties as well.

Interviewer: So do you feel like the difference between military and civilian interprofessional health care teams, it changes based off of the level of care? Or do you think that that mission focus is relevant to every level of care in the military?

Interviewee: Yeah, it's relevant to every level of care. In fact, if you bring - the reservists are a great option - you bring the reservists in who work in the civilian setting, and they perform phenomenally when in that very focused military setting, when the mission's right in front of them.

Interviewer: Right, okay. So we'd like to understand that the kind of health care work that military interprofessional health care teams deliver is different from the work that interprofessional teams do in civilian contexts. Is there any difference in the kinds of work that we ask these military teams to do that we do not expect of civilian teams?

Interviewee: I mean, fundamentally, complex patient care is complex patient care. So whether it's a sick patient in the ICU at a civilian hospital or *[military hospital]* or downrange, the medical principles are all the same. The difference is our "ask" for folks to do that in the most extreme environments. And that just takes relative – that takes a level of training, ingenuity, and flexibility that the military – the construct of what the military brings to the table cannot be reproduced by pretty much anyone else.

Interviewer: Okay. So I'd like to ask you to describe the different health professionals that work in military interprofessional care teams. We understand that there are some kinds of health professionals who are unique in the military context. That is, there are some health care professional roles that are part of military teams but not civilian teams. Can you describe those unique military health professionals?

Interviewee: …obviously there are things like undersea medicine, which are unique to the military. No one else has that mission. I'm just not a

subject matter expert in the area… Undersea medicine… submarine or, like, diving medicine. Or even in a submarine. Diving medicine, there is diving medicine that obviously happens with oilrigs and the like. But medicine in a submarine is fairly unique to the military.

But with respect to *[healthcare profession]*, again, most of the principles are the same. At the margins, there are some differences – for example, the Air Force has the CCAT teams, the Critical Care and Air Transport teams, where you've taken critical care that's delivered in an ICU setting and you've added onto that multiple patient movements, so patient transfers, and doing that in an airplane at altitude, with all the things that come with that. Again, that goes to that adaptability that we talked about. But, again, mostly, the principles of care remain the same whether you're doing it in a tent or in a large facility.

The other area that's emerging that is also extremely unique is far-forward damage control surgery and resuscitation. These very austere surgical teams, oftentimes as little as five folks, that go out and provide damage control surgery or damage control resuscitation, again, taking the principles of taking care of the patient anywhere, but doing it among the most extreme environments. And pretty much no one else in the world does that.

Interviewer: So I'd like to ask you about the dynamics of military interprofessional team collaboration. In the military, not only do we ask health care professionals to work across professional differences – that is, for example, we ask physicians and nurses to work together – but we also ask these individuals to work across the boundaries of rank. That is, sometimes a physician is a major who's working with a nurse who's a colonel and so is the highest-ranking officer on the team. Can you describe how this impacts the collaboration of the care team?

Interviewee: Well, it can't. So I mean, it's a nonstarter if rank trumps clinical expertise in the care of a patient. And for the most part, it doesn't. Everyone recognizes that with respect to a particular problem, the expertise may lie in a person of lesser rank. Doesn't mean they're of lesser ability or lesser skills. The aviation community does this with pilots: who's the lead pilot versus the supporting pilot. So I think that is well recognized, and when folks cross that line, they cross that line at the peril of the patient.

Interviewer: So what do you think a care team would need to do if rank was becoming an issue? How should it be handled?

Interviewee: Well, there's always a leadership structure in any of these settings. Leadership needs to step in and, basically, squash it.

Interviewer: Okay. Good answer. So now that we've had a chance to discuss the differences between civilian and military health care teams, and we've discussed the characteristics of what makes military care teams unique, I'd like to now ask you to reflect on why those differences exist. So during our conversation so far, I've taken a couple notes. You've really remarked that in order for a team to be successful, you highlighted individual currency and competency; awareness of the environment and how that's more critical depending on how austere the environment is; the roles of the other individuals; roles of yourself and what your role is on that team; everyone needs to be trained to a baseline of currency and competency, not just routine care but also in the expeditionary environment; there's a level of just-in-time team training; familiarization of the environment in individual roles; having a well-defined mission, especially in the austere environment; mission focus; effective communication that's multifactorial; and training, ingenuity, and flexibility to deliver care in austere environments. Is there anything that I missed?

Interviewee: Nope.

Interviewer: Great. So with that list in mind, can you tell me why those characteristics are important for military interprofessional health care teams? So why are the characteristics that you've listed as successful important for our interprofessional health care teams?

Interviewee: 'Cause we have a no-fail mission.

Interviewer: A no-fail mission?

Interviewee: No-fail mission. Take care of our nation's heroes. We can't get it wrong. And we need to provide excellence regardless of the setting.

Interviewer: How do you think that affects the team dynamics?

Interviewee: I think most teams recognize that, or if they don't, they need to recognize that. And I think it ups everyone's game.

Interviewer: So thinking back on all your experiences, can you explain to me why the US military needs interprofessional health care teams that are specifically trained to work in the military?

Interviewee: Because no one else can do that mission, in those environments we talked about. Providing care at a fixed hospital in the United States, many – we do it as well as the best, but there are others that do it the same. I would put us in the top tier of health care systems, given what we provide; but, again, there are other top-tier health care systems. But in the expeditionary environment, no other group can bring the ethos, the logistics, the training, and the focus to the mission.

Interviewer: Why do you think that is?

Interviewee: 'Cause it's no one else's mission. So take Exxon Mobil, for example. It's a reasonable example. They are a large multinational company. They do things in extreme environments – oil wells and crazy places. But there's no Exxon Mobil health care system. They outsource it. They outsource it because they don't have the mission that we have, which is to not only do that worldwide mission – we deliver medical care in extreme environments, but we do it in combat, and we do it in support of our nation's heroes in a no-fail way.

Interviewer: So what do you think the military should do to prepare its health professionals to work in these military interprofessional care teams?

Interviewee: So I think we need to address the baseline currency and competency. We actually have a – it's one of the programs that we have in clinical readiness to ensure that for members of the combat casualty care team. That's number one.

It needs to ensure that their pre-deployment practice is robust, that they're taking care of sick patients and using the full range of their skills. We need to do realistic and reasonable pre-deployment team training practice – meaning, essentially, not eight months before a team deploys, practice together, because it probably won't be the same team, but perhaps on the way or when folks – on the way to where they're gonna be deployed, or when they get there, some team training where folks understand the environment, the limitations of their environment, and the roles and responsibilities, as we talked about before. But don't make it so onerous that it detracts from the deployment itself.

Interviewer: To what extent can we prepare these teams? 'Cause you remarked earlier that being in these austere environments is unique to

military interprofessional care teams, so how can we prepare them to that extent here in garrison?

Interviewee: As I mentioned, we're trying to do this with our clinical readiness program. It's a combination of knowing the knowledge. So there's a set of knowledge you need to know to be functional in that austere environment. Fortunately, much of that knowledge has been codified, especially in our world of combat casualty care, through clinical practice guidelines. So basically, we've done some really nice work to distill that and then to have a way to assess that knowledge piece.

There's skills that are a gap between standard environment and the expeditionary environment. So, again, codify those skills, assess those skills, and then train or retrain folks that have a deficit in knowledge or skills. And then, finally, the ability to provide a robust pre-deployment practice, which we've also worked on a metric or a way to do that.

Interviewer: So I'd like to ask you about the ideal structure that you would envision for a military interprofessional care team. In other words, if the grass was always green, money was never an issue, how would you structure a military interprofessional care team to achieve maximum success?

Interviewee: So you would, number one, as we talked about, ensure the currency and competency of every individual member of the team, both in the routine care setting and the expeditionary gap. You do that gap through this approach to assessment of knowledge, assessment of skills, and extracting the readiness value of that routine practice and measuring it and managing to that. That ensures all the individuals are ready.

Then you basically need to build a workforce management plan that maps the expeditionary need, i.e., how many surgeons, medics, anesthesia providers, and so on and so forth, are needed at every level of care. You map that back to making sure that you have enough of those individuals in your inventory.

And then in your – once you effectively deploy those individuals – and we need to be much smarter about how and where and when we use those individuals. But once you effectively deploy those individuals, you tailor their pre-deployment training to be as focused and as impactful as possible. And an element of that should include getting the team members together, familiarizing

with the environment, the communication, everything that we talked about.

Interviewer: So I heard you say we should be smarter about getting these teams prepared to give that. In what ways do you think that we could be better?

Interviewee: We spend a lot of time in pre-deployment training, training to things that perhaps don't have a high return on investment. And oftentimes the pre-deployment training becomes the most onerous element of the entire deployment. For example, we'll send teams to do combat skills training when they're going to a large base where they're really not going to be using those skills effectively. We try to use a one-size-fits-all approach to pre-deployment training, and that's probably not the most effective use of their time or their resources. And it detracts from time that could be spent doing the critical team training that's relevant to our particular mission.

Interviewer: Okay. So I only have two more questions. I'd like you to think back on all your experiences in health care. Can you tell me what makes a military *[healthcare profession]* different from a civilian *[healthcare profession]*, and why is that difference important?

Interviewee: So what makes a military *[healthcare profession]* different than a civilian *[healthcare profession]*?

Interviewer: Mm-hmm.

Interviewee: Number one, the mission focus. We're here for a very precise mission, although the majority of civilian *[healthcare profession]* also have a very similar mission and a similar ethos. So I think that's – the mission's a little different, but not that tremendously different.

I think having the ingenuity to be successful in those austere environments, although through our experience with the reserves is that you take a civilian *[healthcare profession]*, basically make them understand the mission, give them the appropriate training around what military is like, and that individual will succeed just as if they were an active-duty person. In fact, we've been able to leverage our shared ethos with *[healthcare profession]* in order to have a full partnership with our leading organization in *[healthcare profession]*... And I unfortunately think that's fairly unique to *[healthcare profession]*. I don't think that other professional communities share that very tight common bond.

I do think the one thing that makes us – that does let us stand out, and the civilian community has recognized it, is, when our community of *[healthcare profession]* leads, we can build a system – we can do a systematic approach delivering care across facilities that very few other systems can do, and that's been recognized by the civilians as well.

Interviewer: Can you expand on that?

Interviewee: Yeah, so for example, through the foresight of some key *[healthcare profession]*, folks like *[name A, name B]*– those are an Army and an Air Force *[healthcare profession]*– who developed the foundation of what became the Joint Trauma System, we were able to develop a learning health care system that delivered – that led to the driving down of mortality rates despite the injury severity getting worse, in a way that cannot yet be replicated in the civilian sector.

Interviewer: So last question. Based on your experience and this entire interview we've had so far, how would you define a successful military interprofessional health care team?

Interviewee: One that continues to drive innovation and outcomes. So I look at complexity and readiness and quality and outcomes. We need to be able to do the most complex things to be ready, and we damn well need to be the best at doing it. If we meet those two marks, we win.

Interviewer: So before we end the interview, I wanna make sure you had a chance to share all your thoughts and opinions. Is there something we didn't talk about that you think is important, or anything you'd like to add?

Interviewee: Nope.

[End of interview]

Interview B

[Redacted to protect participant anonymity]

Interviewer: Great, so one of the major objectives of our study is to describe the characteristics of a successful military inter professional healthcare team. To that end, we'd like to ask you about what a successful team means for you. So in your opinion, what are the characteristics of a successful military inter professional healthcare team?

Interviewee: My opinion – it starts off with a common goal or shared vision, shared mission. It doesn't matter – I mean, great leadership is a characteristic of course for a successful team. You can be a great leader, but if your team isn't on a shared mission or a common goal, you're herding cats. They could all be well-performing cats, but if they each have their own agenda or their own goal, then in my personal opinion, it doesn't matter necessarily how successful your leadership is until your leadership can bring them all on that common goal.

And so you don't necessarily have to have the best of the best on your team. My personal opinion, but if you have a team that's committed to each other, sharing a common mission, common vision, common goals, with strong and inspirational/influential leadership, those are all the characteristics that I would define as a successful team.

Interviewer: Do you think it changes based off of the context? So these characteristics you've given of team success, do you feel like that changes based off of whether your experience is at a role two facility in *[deployment location]* versus role five here in the US? Do you feel you carry those same characteristics of success or do you feel like they're context dependent?

Interviewee: Actually, not these specific characteristics that I described. I feel they could be applied at multi-levels that they're not context dependent. I don't personally feel that way. I feel… Now each depending – when you're addressing context dependent, I think there's maybe some sub-leadership characteristics that would lead to a successful team. To me, my personal opinion is those are broad.

If you were looking at, for example, a recipe, those are the general broad ingredients that are absolute musts to have a successful dish emerge. You can change it up a little bit, you can throw some nuts

in and maybe some different spices and things like that that might alter the recipe slightly depending on what exactly you're looking for. To me, my analogy would be if you were to use these foundational ingredients required to make that end product that you want. Without them, you're not going to have the end product that you're looking for.

Interviewer: I heard you say certain sub-characteristics of leadership and of teamwork dynamics in certain contexts. Can you elaborate on some of those more specific characteristics for maybe your role to experience versus role five?

Interviewee: Certainly. In my role 2 – so, depending on when we were talking about sub characteristic or the context, depending on who I'm interacting with, I may need to incorporate different types of leadership characteristics. For example when interacting with some of my surgical techs, whether role two or role five, I don't necessarily need to present research-based literature to support my evidence-based decision-making to them. To them, they want to know I'll roll up my sleeves and I'll do the same job that I'm asking them to do.

That I'll get my hands dirty and to me, a sub characteristic would be leading from the front with them or taking charge or demonstrating the same competency, skills and knowledge that they have with them and being able to do whatever it is that I'm asking of them. For example, when I'm leading some of my physicians or my surgeons as the director of operative services in a role five and I tell them these are the clinical practice guidelines that we're going to adhere to as a team, I better make sure that I've got the evidence-base, literature and research to support me when I'm trying to inspire and influence them from a leadership perspective.

I think these little sub characteristics again are – those are context dependent, but your leadership style needs to be slightly modified, depending on who your audience is.

Interviewer: Just taking a couple notes here. So I'd like to ask you to think across the care teams that you either participated in as a healthcare professional and/or the teams you oversaw as a leader. Can you tell me a story of an event when a military inter professional healthcare team proved themselves to be particular successful?

Interviewee: Yeah, actually, we had a patient come into the operating room in *[deployment location]* with a mortar tube in their thigh. Everyone

on the team's initial thought was "we have to amputate." Rather than jumping to the initial decision of amputating this American's leg, the teams took a step back and we started evaluating different courses of action. What if we didn't have to take the leg? What could we do here? What options are available to us? Why don't – rather than looking at the scenario as in we have to amputate, why don't we change our perspective and look at the scenario as a team and what can we capitalize on everyone's skills here?

What can we bring to the table? What can everyone – what special skills – what can we bring to the table that would actually result in a better outcome for this person? So let's, rather than looking at it from the most detrimental outcome, let's look at this and how we can solve the problem for the most beneficial outcome. Once we, as a team, took that approach, and we evaluated different options, we called in all the different specialists and we capitalized on everyone's skills... Sure enough, we were able to remove the tube and save the leg of that American and send them home on two legs. That was pretty inspiring to watch that all occur.

Interviewer: I hear you saying "capitalizing on individual skills" really in that story. Are there any other labels for that situation or characteristics of the team that you would create to -

Interviewee: Absolutely. There was open communication and where there wasn't open communication, the leader of that team went and actively sought open communication. For example, one of the surgical techs, you could tell had some thoughts into how to approach the treatment plan, but yet was hesitant considering the audience to offer their thoughts and experience behind it. The leader of that team picked up on that and saw that and actively went and sought "what do you think, what is your experience?

Tell me – I want to hear from you. What do you think?" That surgical tech was actually able to provide some valuable insight on what they had done in the past and their experience and what they had seen be successful. Again, it was that open communication. Everyone felt equal to each other. No one was made to be put on a higher pedestal than another. And then again, when open communication didn't necessarily occur, it was sought after. If it hadn't have been sought after and that surgical tech might not have been feeling as able to actively voice their opinions and experiences, we might have missed out on a very important aspect of saving this American's leg.

Interviewer: That's incredible.

Interviewee: It was awesome.

Interviewer: Can you tell me what makes inter professional healthcare teams that work in the military different than those that work in civilian settings?

Interviewee: This is a personal thought of mine, which has always been interesting. Sometimes, this can be a restraint that I think in this case it's actually a benefit is the ranking structure. Because for example, … if … rank put *[a nurse]* in charge as a director of surgical services for a bunch of physicians that were less rank than *[the nurse]*, … from this aspect of this inter professional team, *[the nurse]* was put in charge of the leader as a nurse because of *[their]* rank.

And in a civilian sector, that might not have been the case, because of the hierarchical nature that involves medicine sometimes and not saying that's necessarily a negative thing, but at times, you – I've seen in civilian sectors where not the right person has been put in the right job because of a hierarchical nature. On the reverse, that can be a negative in the military, because people are put into leadership positions because of their rank. Not necessarily because of expertise, their leadership potential, or things like that.

So there's definitely some pros and cons to that aspect, but I think also given the nature of the military service that we're soldiers first. At least that's the mentality that I have and mentality that I've seen from a lot of my colleagues is that we're soldiers, we're a part of a bigger team. It's not I'm a doctor, I'm a nurse, I'm a tech. We're soldiers, we're a healthcare team and oh by the way, I provide nursing care.

Interviewer: How would a successful team handle a situation where rank and professional hierarchy aren't aligned?

Interviewee: I've seen – it's definitely a challenge. I have seen where teams have not been successful, because of that misalignment. I have seen – there's definitely tools that the DoD is trying to put in place. For example, in operating room, there's Team STEPPS that's put into place to try to alleviate that misalignment sometimes where everyone is supposed to feel supported and able to speak up when need be. But I think that's still a current challenge no matter what the environment, whether civilian or military. That's still a challenge, but I see it as a challenge that's slowly being eradicated. Slowly, but I feel like we're starting to get there.

Interviewer: Do you – are there any other insights that you can provide on what we expect of military inter professional teams versus civilians? Any other differences you see?

Interviewee: Not that comes to mind, only because for me, all of my medical career has been in the military. So my civilian experience has been through occasional clinicals, whether I was in some type of school, whether undergraduate or graduate. But to really have a broad understanding of inter professional teams within civilian sector, it's hard for me to articulate that a little more, because I just don't have the experience with that.

Interviewer: Right, that makes sense. I'd like to ask you to describe the different health professionals that work in military inter professional care teams. We understand there are some kinds of health professionals who are unique in the military context. That is there are some healthcare professional roles that are part of military teams, but not civilian teams. Can you describe those unique military health professionals?

Interviewee: Let's see. Okay, one that comes to mind is – I don't know how much of this in the civilian sector actually exists, but unfortunately, these guys get forgotten, as part of the inter professional team a lot, but our biomedical military, and they come to mind, because in the civilian sector – I'll use an example of an operating room piece of equipment like an arthroscopy pump or something, which is a pump that pumps large amounts of liquids into a joint for an orthopedic procedure.

So to my knowledge in the civilian sector, let's say your pump goes down. You call that company in. We'll use the example *[name of company]* or something. You call a *[name of company]* rep in, the *[name of company]* rep does the work on the piece of equipment. In military, that's not the case. Pump goes down, the piece of equipment has to go to biomedical and you usually have civilian or an enlisted technician, which works on that piece of equipment. So the enlisted technicians have to be jacks of all trades when it comes to the equipment in a hospital. That can actually make or break patient care. If we don't have a piece of equipment that we need to do to take care or to do surgical cases and then we can't take care of our patient and we have to refer them out, there's a whole trickle-down effect.

But then, when we're looking at bringing new equipment in, you know, as an inter professional team for example, let's say we want

to bring in the latest and greatest toy to do patient care to improve patient outcomes, well we never consult biomed. We never bring them in to say what are your thoughts? Should we get a contract with the company to repair this? Are you guys able to support this? I use this example based on my previous experience as *[redacted to protect participant anonymity]*.

That's a position on the team that doesn't get incorporated as often as it should and a position that I don't see being used within the civilian sector. Mostly because civilian sector will go straight through a specific company for a piece of equipment.

Interviewer: Do you think it's a positive contribution to the team keeping it within the military?

Interviewee: Personal opinion, yes and no. I think that it's great to have the resources there and available when you need them. Because if they're familiar with the equipment and they really know the equipment then yeah, they're there and they're available and they can help get things back online. But again, they have to be a jack of all trades. So a jack of all trades is the master of none and so yeah, it could be difficult especially when the original path – the better pathway was just to go straight to the company. So I definitely see it as there's pros and cons associated with it. Do I take a strong stance either way? No, not necessarily. Mostly because I've just been so used to it my entire military career that I haven't necessarily formulated a strong tendency towards yay or nay when it comes to that position of a team.

Interviewer: What are your thoughts on – because these are enlisted folks, right, that are taking on these jack-of-all-trade positions? What are your thoughts on the enlisted healthcare role also in your role two experience in *[deployment location]*?

Interviewee: Can you repeat that, I'm so sorry.

Interviewer: That's okay, the enlisted health professionals - what is their role and their contribution more in theater. For example, when you were in *[deployment location]*, did you have any medics or enlisted folk out there and what was your opinion of how they contributed to the team?

Interviewee: Absolutely. So I – my NCOIC that worked with me, especially in theater, was my right hand man. I find their roles to be extremely valuable. I think they're experienced, they're knowledged, they bring a different perspective and -

Interviewer: What perspective do you think they're bringing that's unique?

Interviewee: *[Redacted to protect participant anonymity]*

You know, your enlisted personnel can come in and say "Hey look, this is a more efficient way to handle it, this is the way I've seen it in the past. It may not be the most luxurious, but this is how we can get the most bang for our buck." So sometimes, they bring a more realistic look into the business of handling things. They look at it more of an efficiency standpoint rather than more of the luxuries kind of behind certain situations…

Interviewer: So do you feel like there are certain roles? You said the biomedical tech role is unique to the military. Do you feel that kind of administrative efficiency role that these enlisted are contributing to in a deployed environment, do you feel that's pretty unique position as well?

Interviewee: Yeah, actually I do, especially in the deployed environment. We still have equipment in the deployed environment that we have to use. The piece of equipment goes down in a deployed environment, we can't call the company in to Afghanistan for them to bring their rep over to work on a piece of equipment. So that's a good point that you bring up is that absolutely in an environment like that, we need that role, because we – like I said, we can't function without somebody who can fix the things that are broken.

Interviewer: Okay, now that we've had a chance to discuss the differences between civilian and military healthcare teams and we've discussed characteristics of what makes military care teams unique, I'd now like to ask you to reflect on why those differences exist. I've taken some notes and during our conversation so far, I've noted that you've really focused on – in order for a team to be successful, they need to have a common goal, a shared vision, a shared mission, and that great leadership – as good as great leadership is, granted, everybody on the team has to have that common goal and shared mission.

Every team member doesn't have to be the best of the best, they just have to have good inspiration and influential leadership in order to succeed. You talked a bit about the sub leadership characteristics depending on certain contexts. Your leadership style has to be modified, so when you're working with med techs, you're more focused on showing that you're willing to get your hands dirty, leading with them from the front. Whereas if you're in

a role five facility, and you're talking to some higher ranked people in a leadership position.

You have to take a different approach in proving that you know what you're doing, you have evidence-based research to back up the things that you're trying to communicate. But overall, as a team, everybody capitalizing on individual skills for the most beneficial outcome, having - feeling of equality throughout the team, and you really also focused on not only open communication, but actively seeking open communication. So you provided that example of one of the higher ranking people on the team seeking out some lower ranking people and saying hey, I want to hear your opinion.

Is there anything I missed? Is there anything else you think really contributes to team success?

Interviewee: No, nothing that immediately comes to mind.

Interviewer: Okay, great. Can you tell me with that list that I just reiterated for you why you think those characteristics are important for military inter professional healthcare teams. Why are these characteristics that you've listed as successful important for our military inter professional healthcare teams?

Interviewee: Let's see, I feel like the characteristics I listed aren't – I mean, they are important for military healthcare teams. They could also be the same characteristics that could be important for a civilian team. But specifically, within a military team, the soldiers are "mission first, people always" and so soldiers need to have - whether enlisted or an officer - I feel like soldiers want to have what is that end goal, what is that end state, what are we working towards? So that characteristic of having that shared mission, that shared vision kind of goes back to my analogy of herding cats.

You need to bring your troops together, bring your team together and in order to do that, everyone has to be on the same page, especially within a military setting. And I say that, but again, I say that speaking from military experience. It's kind of hard to say… I would assume the same could be applied to a civilian, a civilian team as well. But again, the military from day one, it's driven into your head, "mission first, people always."

To me, to have that and to make sure that's clear and agreed upon by that team is going to be your driving factor to what you're

trying to accomplish and to what you're trying to accomplish successfully.

Interviewer: What do you think that mission is in the military? If you say that a civilian healthcare team likely also has a mission focus and a military healthcare team has a mission focus, do you think they have the same mission focus or do you think it's different because you're a military healthcare team and you have a different mission?

Interviewee: In the grand scheme of things, broadly speaking, no, I would say it's the same mission. It's there's different ways of putting that mission. The civilian community wants to provide the best care that they can to their beneficiaries or their patient population. Military wants to provide the best care that they can to the beneficiaries or patient population. So that's in a sense the same mission, the same goal. But how we go about doing that is very different.

I've seen civilian inter professional teams that work like a well-oiled machine, because that team has trained and been together for a very long time. Whereas military sometimes, some of the downfalls of a military team is that it's a rotating door. Once your team gets well-oiled and put together and functioning, next thing you know, somebody's leaving, the next person's coming in and then you're relearning that team.

Interviewer: What do you think a military team has to do specifically in order to keep that team – because you say the teams keep changing, people keep coming in and out of the door. What do you think military healthcare teams uniquely have to do in order to keep those teams running efficiently, since they're always changing?

Interviewee: If we're looking at it from the perspective of just the military, I think it goes back to that shared mission, that shared vision. And the openness and understanding of that revolving door whereas in a civilian community, if someone has to leave and a new person comes in, that can be very earth-shattering and altering to that team. In the military, it becomes a way of life. It becomes the norm, which the military is used to. So without that shared mission, that shared vision, a military team might not be as successful with a revolving door.

Now, the civilian community could have the same shared mission, shared vision, but it may take them longer to come on board with it, because of the fact that if a new person comes into that well-

oiled machine, it could, like I said, have more earth-shattering effects than it would in the military where that's the way of life for us.

Interviewer: And you feel like it's that shared mission that kind of brings the team together. Is that correct, me saying that?

Interviewee: Yeah, definitely.

Interviewer: Great, so thinking back on all your experiences, can you explain why the US military needs inter professional healthcare teams that are specifically trained to work in the military?

Interviewee: Because – so not only – so military medicine is unique and not only do we have to do all the same jobs as a civilian inter professional team. But now we have to do it understanding and knowing rules and regulations of the military. Not just the military, but DoD. As an army inter professional medical provider, I have to know army rules and regulations. But oh, by the way, I may have a navy and air force colleague on the team with me now as well.

I need to have an understanding of their rules and regulations as well. We basically take two full-time jobs and we make it one full-time job. Not only do I have to be a medical professional as a full-time job, but I'm a service member as a full-time job. I say service member rather than a soldier, because the service member, which can then essentially be three full-time jobs, whether army, navy, or air force is now one full-time job as a service member. So we have to take two worlds and blend it into one.

Interviewer: What do you think the military should be doing to prepare its health professionals to work in military inter professional care teams?

Interviewee: In the light of the notorious phrase of going purple, I think a big component, we do an excellent job in making sure that our healthcare professionals are educated from a healthcare perspective. We do an outstanding job of that.

There's no doubt or question that I would have that our inter professional teams couldn't come in and deliver safe, high quality healthcare. What I feel like our new challenge is for a military inter professional team is now becoming the purple, now taking that blended service and having an understanding of what each service brings and capitalizing on that. The navy obviously knows how to do medicine on the water.

Air force knows how to do medicine in the air. Army knows how to do medicine on the ground, but now those teams are all blended together. So how do we now train our inter professional teams now to work in a tri-service environment. I think that's a huge aspect of inter professional teams that we're not quite addressing yet, as we're transitioning into this new era of military IPE.

Interviewer: Going off of that, if you had to think about the ideal structure that you'd envisioned for military inter professional care teams, so if grass was always green, money was never an issue, how would you structure a military inter professional care team to achieve maximum success?

Interviewee: That's like, what would you do with a million dollars? Oh my.

Interviewer: I heard you say joint, the whole purple perspective. I hear you saying essentially getting all healthcare providers on the same page in terms of all three branches, if I'm correct in saying that. Can you expand further?

Interviewee: If I could provide – if I could take an IPE team and take them through, if I had the time, manpower, resources, all the money, take them through tri-service healthcare operational training. So putting the army and navy up in the air, putting the army and air force out on the sea, bringing the air force, the navy onto the boots on the ground.

So really for an example here at the *[location]*, we're trying, for our specific program, we're trying to get our students to go do some exercises out on the *[name of ship]*. They're never going to – the *[name of ship]*. They're never going to have an opportunity to do something like that. We're trying to get them in with the air force to do in route critical care. You never know when you're going to be down range or something and someone's going to grab you and throw you in the back of the C130 and tell you, you need to take care of these patients.

When we talk about operational readiness, in my opinion, that's a huge definition. It's not only able to do your clinical skills in your clinical environment, but it's able to do it in multi environments with multiple services. Yeah, and then not only having exposure for that individual healthcare provider and different operational environments, but now putting together the team and bringing the team together to function in all those different operational

environments I think would really set our military IPE teams up for better success.

For example, when I was in *[deployment location]*, we got used to the luxuries that our immediate safe haven environment provided to us. We got used to – we were able. It became in a sense kind of a ground hog day, because we knew where our supplies were, we had the same setup, same equipment. But let's say heaven forbid we were mortared and it took out our entire hospital there. How would we re-rally and refunction now and could we continue patient care? Would we still be able to take care of our patients in a very different environment? I don't think at the time we would have been prepared to.

Interviewer: Okay, I'd like you to think back on all your experiences and healthcare. Can you tell me what makes a military *[healthcare profession]* different from a civilian *[healthcare profession]* and why that difference is important. I know you've said it's hard for you to compare, because you haven't had much civilian experience. Anything you can draw upon just from recognizing this is unique to the military, for example.

Interviewee: Absolutely I can, because although I haven't had much experience in the civilian sector, a lot of my friends I went to school with are in the civilian sector. I have been put into some very big leadership roles. Very – in a sense - early in my career, compared to my civilian counterparts for *[healthcare profession]*. I still have friends – I've been a *[healthcare profession]* for coming up on *[number]* years and I still have friends that are just working as clinical staff *[healthcare profession]*.

I have already been director of *[title of department]* for an entire hospital. Within six months of becoming a brand new *[healthcare profession]*, I was made *[title of role]* of the unit that I was working on by given rank, which I think has helped mold me and provided leadership experience. Of course there's been times that it's been scary and you feel like you've kind of been thrown into the fire.

But I think being an army *[healthcare profession]* has afforded me and provided me far more leadership opportunities than I would have if I would have stayed a civilian *[healthcare profession]*.

Interviewer: Do you think it's because of ranking systems in the military that it increases your leadership potential for different positions or is it also just because you're a military *[healthcare profession]* and

they tend to give more leadership to military *[healthcare profession]*?

Interviewee: Some of it is rank, but also I think the military has shifted to where we're doing a really great job with talent management. I was given the director of *[title of department]* based on rank, but I was also junior in rank compared to some of my competitors. I was a rank or two below some of the competitors that I beat out. Again, it was based on rank, but it was also talent management. So I was – even though I was junior rank to some of my competitors, the individuals that selected me decided that I was best qualified, credentialed, and skilled to take on that role than those of higher rank than me.

Given leadership potentials and opportunities that I had been given prior to the military…

Interviewer: …As a followup question, do you think that kind of the freedom the military provides to give you more leadership positions and give certain members more responsibility, do you think that impacts how these inter professional teams work together and enables their success? Do you think how the military is unique in giving these leadership opportunities has a positive or negative effect on how our teams collaborate?

Interviewee: I think they can – I think both. I think that – I'm sorry, can you repeat that question one more time for me? I want to make sure I articulate it appropriately.

Interviewer: It's completely fine. You were discussing how you feel that both the rank in the military, but also being a military *[healthcare profession]* has given you more leadership opportunity, because the military is unique in giving those options to military *[healthcare profession]* or military *[healthcare profession]*, et cetera. Do you think that impacts the inter professional care team when everybody on a team working together inter professionally, the fact that certain team members have been given more leadership positions or more responsibility, you know, which is different from a civilian inter professional care team. Do you think it impacts our military interprofessional teams in a positive or negative way?

Interviewee: Yeah, absolutely. So, kind of going back to what I was saying earlier about talent management, given where I'm currently at, if I was plucked from where I'm at and I was put into a different IPE and – or inter professional team - and my past experiences, my

knowledge, the leadership opportunities afforded to me, I would hope would be beneficial to that team. But in hindsight, I know a lot of – unfortunately, with the good, there's the bad.

I know a lot of officers that were put in leadership positions, *[healthcare profession]*, based on their rank and aren't necessarily a leader. They were just put in that position given their rank and so they haven't had – although they've had "experiences" put in there, doesn't mean they're necessarily a good leader or a good member of an inter professional team. They may, on paper, have experiences, but if put into an inter professional team, because of their rank and the fact that they were forced, voluntold to have experiences doesn't mean they're necessarily going to be a good asset to that team.

But it's one of the catch 22s of the military is that the longer you stay in, of course, the higher you go in rank. You may or may not be put into – electively put into leadership position and you may or may not be good at it. So although on paper you could have a lot of experience, given the nature of the military, in reality, you might not be a successful leader or a successful inter professional team member.

Interviewer: Based on the conversation that we've had so far and the ideas that you've generated about team success, can you give me a definition of a successful military inter professional healthcare team?

Interviewee: I would say - I don't know if I'm going to articulate a verbatim definition that's going to come out right, but I'm going to string a couple of thoughts together.

Interviewer: Perfect, that's all we need.

Interviewee: I would say a successful military inter professional team would be one that would be trained and successful in accomplishing their mission with or without the presence of their designated leader. Again, I've always been told if your team doesn't need you, then you've trained them right. You've done a good job leading them. And I say successful and with successful, because success can mean different things in different contexts. Successful as in whatever their mission is, whatever their goal is, achieving that.

And then in that, it can be efficient, effective, safely, you know, all of those positive attributes and characteristics that encompass a well-functioning team. But successfully achieving their mission, their goals, objectives in the best methods or the best or most

appropriate I guess you could say. My biggest thing is whether – that it could be done and accomplished with or without the presence of the designated leader.

Interviewer: That's interesting. Can you expand more on that? What characteristics would a team have, if they can succeed without a leader? Why are they succeeding?

Interviewee: When I say without, I mean the physical presence. What I'm envisioning in my head and what I'm seeing in my head when I say that is - so let's say we're training up to deploy and function as a forward surgical team and we have our designated leader. Let's say we're down range and we have a mass cal happen. And the designated leader can't physically be there for whatever reason at that moment. But the team that is there comes together and is successful.

All the care that they're giving to the patients and everyone, they have a successful response to this mass cal and then a leader didn't need – this designated leader, this FST commander, whomever, didn't have to actually physically be present in order for that team to be successful. That's kind of what I'm envisioning when I say that. The leader has inspired and has trained the team well enough that they can physically do their job without the physical presence of the leader there.

Interviewer: What do you think the team is doing without the leader there, what are they doing that's letting them be successful? What characteristics are they showing?

Interviewee: I think it goes back to a lot of the ones I originally talked about. That shared, that communication, that open communication with each other. Shared roles and responsibilities and then having an understanding of that, too. So if I know my role and responsibility is that of *[healthcare profession]* and I know what that entails, then I better be able to perform that and perform it well and appropriately. But then also, I know the roles and responsibilities of my colleagues, so I can support them.

Or if they're falling or they're faltering somewhere, even if it's outside of my scope of practice, I can at least be aware of that and notify someone to support them as well. So it's that inter connection of the team, that knowing my job, but knowing the jobs of those around me. If they're not successful at their job, I can intervene to an extent.

Interviewer: Interesting, okay, so I want to make sure you had the chance to share all your thoughts and opinions. Is there something we didn’t talk about that you think is important or anything you’d like to add?

Interviewee: Not that I can think of off the top of my head, no.

[End of interview]

Interview C

[Redacted to protect participant anonymity]

Interviewer: So as for the interview questions, one of the major objectives of our study is to describe the characteristics of a successful military interprofessional health care team. To that end, we'd like to ask you about what a successful team means for you.

First question: what are the characteristics, in your opinion, of a successful military interprofessional health care team?

Interviewee: Of one that possesses mutual respect between members, that are respectful, that each can contribute to the clinical process outcome that you're seeking, and that, by that token, although there is leadership of that team, that that leadership is respectful to the participation and contribution of all members.

Interviewer: So as a follow-up to that question, do you feel like that definition you gave me about mutual respect between all members is particular to a specific level of care, or do you think that the dynamics of what makes a team successful and what characteristics create a successful military interprofessional health care team change based off of the level of care, meaning if you're in theater versus in garrison?

Interviewee: No. I think it's the same.

Interviewer: You think it's the same. Okay.

So I'd like to ask you to think back across the care teams that you either participated in as a health care professional and/or the teams you oversaw as a leader. Can you tell me a story of an event when a military interprofessional health care team proved themselves to be particularly successful?

Interviewee: Yeah. I can relate to you the story of a grievously wounded service member that came in to the ER of a combat hospital. We had run out of all the sort of officially trained people to really interact with that casualty, just because of the casualty flow. A number of the members of the walk-in ambulatory area were available and contributed to the resuscitation of that casualty and, as such, I remember seeing one of the patient registration members with her hand inside the chest of this injured service member holding onto a blood vessel in order to give him a chance to survive.

Interviewer: Wow.

Interviewee: She happened to also be a medic, but had been assigned as a PAD member. Well, the mutual respect and understanding of each other understood that this was a person that could do additional work and was given the opportunity to do so. And so that's that.

In those type of situations, I think you have to develop a respect, and you have to break the cultural prejudices of people. Let me give you an example of that.

One of the reasons we've been so successful in resuscitation of seriously wounded casualties is that we've used some of the British models of resuscitation... It took about a week for us to sorta break the prejudices of our surgeons. Once we began to do so, we were able to follow a British model of resuscitation, which was much more advanced in fluid resuscitation than we had in America today.

Now, part of this is the evolution of American health care training. I view us very similar to where we were 40 years ago or so with pilots, where pilots were god and everybody else just sort of responded to what the pilot said, and we used to crash airplanes like crazy. But now we've moved to much more of a team-based, safety-based system, where any member of the team can interrupt the process in order to deliver reliability. And the outcome of that process, we crash a lot less airplanes.

My thought is, when you begin thinking about interdisciplinary health care and health care teams, you must move them to that kind of model where everyone is empowered to move to the outcome that you want. And whether you're dealing with a pharmacist or an anesthesia provider or a surgeon, they all get equal say.

Now somebody's gotta be in charge, but you have to begin to break the cultural prejudices of classic American and western physician training in order to get to that point.

Interviewer: So what I'm really hearing is leadership is necessary. Granted, when I hear "breaking cultural prejudices," are you talking about in terms of professional hierarchy, correct?

Interviewee: That's exactly what I'm talking about.

Interviewer: Okay. Perfect. Thank you.

Can you tell me what makes interprofessional health care teams that work in the military different from those that work in civilian settings?

Interviewee: Yeah. I think we're overwhelmed sometimes with our rank structure, and that rank structure creates an additional overlay to prejudices that are barriers to success. I would consider it a negative.

Interviewer: Really? Can you give me a story or an example of how rank would be a barrier to patient outcome, or whatever you see as success?

Interviewee: I think that very often in high stress situations you'll see somebody coming in and sort of pulling rank in that process, not allowing the team to do its good work. I think highly cohesive teams need to be fairly autonomous from the rank structure. Literally, you have to get people to go into – almost like they do in an AAR situation.

So you try and get people to go – and when we go into our after action reviews, people sort of take their rank off in order to get honest communication. That's what you have to get people to. It has to be a rank-indiscriminate.

Remember what you're doing. This is all patient-focused. If you're patient-focused, and this whole group descends upon the patient, it should be rank-agnostic for the benefit of the patient.

Interviewer: Interesting.

Interviewee: Now, on the civilian side I never had that problem. When I ran civilian health care systems, it was really more the fact that I had a sort of classic hierarchical system in which the doctor was god and everybody else needed to do what the doctor said, even if the doctor wasn't the expert in that area.

Interviewer: Did you see that as a contributor to the success of the team, that there was a professional hierarchy there in the civilian contexts?

Interviewee: No. I always considered it a negative. I always considered it a negative, and I still think that, in order to deliver high reliability outcomes and to reduce error, you need honest communication between the team, and you need to empower everybody at the same level.

Interviewer: So I feel like I'm hearing that it's not only military rank structure as an issue but just basic medical, professional hierarchical rank

structure in the civilian sector also creates an issue in team success. I'm hearing both. Am I correct?

Interviewee: It does. Correct. But remember, in the military you have both of those.

Interviewer: Right.

Interviewee: And so your initial question reflected sort of how did I compare them. I think it's more difficult in the military because you've got this overlay of rank also.

Interviewer: So what do you think that – 'cause one of our – I'm gonna skip to one of our later questions – 'cause we talk about the dynamics in military interprofessional team collaboration, and not only do we ask health care professionals to work across professional differences, for example physicians and nurses, but we also ask those individuals to work across boundaries of rank. How do you think that this impacts the collaboration of the care team in the military setting that we have both of these kind of rank issues, both military-wise and professional hierarchy? How do you think that impacts the care team?

Interviewee: I think it's a potential negative. It must be recognized in order to have it be overcome.

Interviewer: Okay.

Interviewee: For instance, mass casualty situation in *[deployment location]*, multiple patients. We always placed a single leader in the center, at one end of the care area as we were triaging patients, who made all final decisions on where pieces of the team went to, that literally was unencumbered by care, had stepped off to the side unemotionally, and would stand and just sort of watch what was going on at each table. The lead at each table would talk to that coordinating person to decide who went to the operating room first, who didn't, what – but was unemotional in the resuscitation.

The problem you have in resuscitation in combat situations is virtually all of the casualties have an emotional attachment to the care team, so it's impossible for you to emotionally detach yourself because it is another service member, or you know the person, depending on the size of the unit. It's very difficult to unemotionally make decisions. Therefore, we always put somebody in charge to sort of overwhelm the rank structure and leadership.

Now we have a lead at each table, but it was always a multidisciplinary approach. At times the anesthesia nurse took precedence over the surgeon. The person doing the resuscitation might not necessarily be a surgeon because the surgeons were all busy in the operating rooms.

Interviewer: Okay. So am I correct in hearing that you see kind of the emotional attachment with military service members and their casualties as a negative impact on the care team?

Interviewee: Yes. I consider it a negative impact because it makes it very difficult to separate yourself.

Interviewer: Okay.

Next we'd like to understand if the kind of health care work that military interprofessional care teams deliver is different from the work that interprofessional teams do in civilian contexts. Is there any difference in the kinds of work that we ask these military teams to do that we do not expect of civilian teams?

Interviewee: Yeah. I think that there is triage work that triages the survivable over the most catastrophically injured in a earlier decision made in those that will not survive on the civilian side, where, just necessarily, the worst would go first. So I think triage is different.

I think triage also is tempered by the availability of supplies, and, therefore, decisions are made that you might be able to save three if you didn't take the one that was gonna use up all your blood supply. So the work is dramatically different than on the civilian side.

I think that the catastrophe of injuries are much different. The concept of an integrated team – that stretches back to the United States – so that you don't have to fix everything at once is part of the staged approach that is dramatically different in the military than on the commercial side.

Interviewer: Interesting.

Interviewee: And so that immediate stabilization surgery in the field may be – is dramatically different than what I saw done in the civilian and commercial side. And so I find the work much, much different.

Just the sheer complexity is rarely seen now. If you're in Las Vegas, and you're attempting to resuscitate 500 shot people, that – or you're in Boston and a bomb goes off during a marathon, it's gonna be very similar. But the decisions in an austere setting where you don't have enough supplies create unbelievably horrific decisions that are far, far away from what people deal with in the commercial sector – and should be, and should be. When you're literally deciding who gets ventilators and who doesn't, those are things that should be a long way away from commercial health care.

Interviewer: So what characteristics do you think that team members in the military that have to deal with these more catastrophic events – lack of resources, et cetera – what characteristics of the team do you think are needed to make them successful in those situations that you wouldn't necessarily need to see in a civilian team?

Interviewee: A cohesive-enough team that is respectful of those decisions even when they seem to be abhorrent.

Interviewer: Okay.

I would like to ask you to describe the different health professionals that work in military interprofessional care teams. We understand that there are some kinds of health professionals who are unique in the military context, that is, there are some health care professional roles that are part of the military team but do not exist in civilian teams. Can you describe those unique military health professionals?

Interviewee: Yeah. On the Navy side, your independent duty corpsmen are dramatically different than on the civilian side. I think that, then, the expertise in aerospace medicine and in human performance are different than on the Air Force side. On the Army side, those individuals – no, I'm not sure those are unique. I don't know. I think that's probably what jumps out at me right now. I think that the ability of nurses to operate independently of anesthesia providers – so anesthesia – CRNAs that operate independently of physicians is unique to the military, although it's becoming more common in some areas of the United States as there's a shortage of anesthesiologists. But in our forward surgical teams has no anesthesiologist.

Interviewer: Interesting. Okay.

So now that we've had a chance to discuss the differences between civilian and military health care teams, and we've discussed the characteristics of what makes military care teams unique, I'd like to now ask you to reflect on why those differences exist.

I've taken a couple notes thus far, and I've noted that the general themes you've been really explaining to me have been mutual respect between team members; respecting that each team member contributes to the clinical process outcome; that although leadership is necessary and important, leadership also needs to be respectful to the participation contribution of all team members, giving opportunity for all team members to contribute whatever skills they have; breaking cultural prejudices so each team member feels empowered to work towards the goal and outcome all get an equal say and role in the team; highly cohesive teams that need to be autonomous from rank – and we said both military rank and also professional hierarchy – honest communication and empowerment of team members; and also cohesive teams that are respectful of whatever decisions are made, especially in these military health care teams that are working in pretty catastrophic situations in theater. Is that an accurate summary? Is there anything that I misinterpreted?

Interviewee: No. You got it.

Interviewer: Okay. Great. So with that list in mind, can you tell me why those characteristics are important for military interprofessional health care teams, so why these characteristics you've listed to me as successful are important for our teams?

Interviewee: It allows you to realize the broad, intellectual, and technical expertise of your team. You can't realize the intellectual input and technical expertise of your team unless you allow them that safety to operate and those sort of rules and parameters that allow the safetyness – that you're leveraging everybody. So somebody's not standing there quietly, saying, "Well, if the surgeon really wanted my input, he'd ask for it," or – and so the only way you deliver full value of the team to the patient.

Interviewer: So when you say "safety," are you saying emotional safety?

Interviewee: Yes.

Interviewer: Okay. Understood.

So thinking back on all of your experiences, can you explain to me why the US military needs interprofessional health care teams that are specifically trained to work in our military?

Interviewee: There are not enough of us, and, specifically, unless you treat people with respect and they feel that they're an important part of the team and have broad control and contribution to the mission, they're not gonna stay with you. They're gonna come for a short time. They're gonna get their training. They're gonna move on because they can make more money elsewhere.

Interviewer: Interesting.

Interviewee: They'll only be tied to the mission if they're treated with absolute respect.

Interviewer: Okay. So what should the military be doing to prepare its health professionals to work in these military interprofessional care teams? And I know you just highlighted respect, really in terms of attrition. Is there anything you could add in terms of preparation?

Interviewee: Yeah. I think the classic licensure-based training, as for physicians and nurses and other health care professionals, needs to incorporate team-based approached training, showing the value of teams, putting them in situations where they show that outcomes are dramatically different when teams work in a respectful and collaborative manner, that there are other models in other parts of the world that are nurse-centric because of the lack of physicians that physicians are actually consultants to the nurse lead.

There are other places where – even in the United States – where pharmacists are leading, and if the outcome – I think *[name]* Clinic has shown that the most important thing for a post cardiac surgery patient or post heart attack patient is a good clinical pharmacist in the three months after hospitalization, who make sure that they're taking their medicine effectively and that that's being worked to appropriate levels without complication, has way higher value than a nurse or a physician with much lower mortality and morbidity.

So I think you have to train this. If you're gonna break a culture, you have to train it. You need to train it, and this also means you have to break the culture of the educational hierarchy, which is where your struggle is. In a place like any university, there is such an ingrained hierarchy in the university, it is really hard to begin to break that. Again, people have taken decades, and its why universities are so difficult to make change. And everybody says,

"Well, we're evidence-based. We're this. We're that," and my answer to that is that if we were truly evidence-based, it wouldn't take us so many years to adopt changes in practice.

Interviewer: What do you think needs to be done in order to kind of disintegrate this hierarchy that we have in our universities?

Interviewee: Inspiring leaders that begin to collect the data that shows the difference. I think as the data comes together of different models, we'll do better. But I think it's this kind of work that you're doing now that begins to get the input of individuals. And I don't know if my input is any different than any of your other collaborators, but it's – to me, it's pretty straightforward. You gotta change the method in which you train physicians, nurses, and ancillary professionals to show a level of equal respect.

Interviewer: Interesting. So if I ask you about the ideal structure that you would envision for military interprofessional health care teams – so if the grass was always green, money was never an issue, politics not involved, et cetera – how would you structure a military interprofessional care team to achieve maximum success?

Interviewee: I'd probably dump it on its head and put a nurse in charge… I think that nurses have a better baseline training and holistic approach to patient care. Physicians are much better at technical interventions and individual technical points, but I think on a holistic basis nurses do a better job. That doesn't mean I'm not a value to the team, but I'd probably dump the thing on its head and really look at other professionals that ought to be sorta leading the team and then engaging.

Now, you could do it differently. I talked to you earlier about the… British.

Interviewer: Yes.

Interviewee: Resuscitation management in the English system is done by the ER doctor, and all fluid resuscitation, and, literally, the surgeon is left to do technical surgical work with the overall care of the patient, with the ER doctor staying right through the surgical procedure at the foot of the bed and the anesthesia provider simply managing oxygenation and the flow of anesthesia products and the management of the airway. So there's a third member of the team, which is the ER doctor, who just carries that patient right through to the ICU, so from the point they hit the door, right through to the ICU.

It's a different model. I think we just have to begin to explode the mess of who oughta be in charge. Today's surgeon would just have an absolute chest pain and stroke over that unless you'd actually work that and saw the fact that the survival of patients was much higher in those sort of models.

Interviewer: Do you think that this is specific to a Level Five facility here in the US, or do you think that this is also true to our deployed teams?

Interviewee: Oh, I think it's true to our deployed teams also.

Interviewer: Great.

Interviewee: When you get down to austere forward surgical team with 20 people, it's pretty hard. That team's gotta work sort of – you know, everybody's doing everything just 'cause they're so austere. But once you get above that to the combat hospitals, you begin to get the ability to begin to work that more effectively and have the technology available to you to make that happen.

Interviewer: Okay.

So we've spent a lot of time talking about success, but sometimes to really understand success you also have to understand obstacles and failures. Can you give us an example of a military interprofessional health care team collaboration that didn't work well? And I know that you mentioned one of the characteristics contributing to this problem being professional rank or military rank. Are there any other characteristics you can think of?

Interviewee: Yeah. I have a number of examples in theater of teams where, because of the autocratic nature of the surgeon, we were unable to reach success and had much higher failure rates. They were so oppressive to the rest of the team, the rest of the team just sorta shut down – did their job, but just shut down and didn't contribute what they possibly could.

And I go back to my concept that there's gotta be an emotionally safe environment for you to take intellectual risk and technical risk in order to deliver maximum value. There's commanders of surgical teams that I relieved because they created an environment that was not appropriate.

Interviewer: Okay.

So I'd like you to think back on all of your experiences in health care. Can you tell me what makes a military *[healthcare profession]* different from a civilian *[healthcare profession]*, and why is that difference important?

Interviewee: Yeah. It's not all about them all the time, and it's not all about – you know, when I talk to civilian *[healthcare profession]*, they are way more interested in being able to take vacation when they want to and do what they want with their lives. What makes people different in the military is they're willing to make their own personal needs subordinate to the needs of the mission and the nation. That's extraordinary. I don't think it has much to do with income. I think it has to do with people's willingness to put something else more important than themselves in place.

I find in the commercial world… I found most of them some of the most narcissistic, self-centered people that I'd ever met.

One of the things I love about government service – and it doesn't have to be uniformed. I find the same thing in government service in the VA and in the commercial – in the civilians that work in our military treatment facilities – these are people that really do get connected to the mission, and they love the mission. They do good health care, but they're willing to accept the fact that they may not get the day off they want, and they may not even be able to take the vacation that they want at the time they want. They are willing to subordinate themselves with that mission.

Interviewer: How do you think that this impacts the dynamic of the care team?

Interviewee: I'm not sure that there's always this much respect for that as there needs to be. I think especially the management teams need to be more respectful of what those teams deliver. I think there is a connectedness, though, at the actual patient care level, and a connectedness to the service of the patient and what that patient represents – that I saw in both my work at VA as well as my work in DoD – that I found absolutely extraordinarily refreshing.

Interviewer: So based off of this conversation that we've had so far, can you give me a final definition on what you feel makes a successful military interprofessional health care team?

Interviewee: Mutual respect, a removal of the cultural encumbrances of their licensure and the level of their licensure from a hierarchical situation – so the abolishment of a care hierarchy – and a culture of emotional safety that allows the broad application of skillsets to the

patients' outcome. And if you really think about this, this is the true definition of what patient-centric means.

Interviewer: Interesting. Okay. Well, thank you for your time. Before we end the interview, I want to make sure you had a chance to share all your thoughts and opinions. Is there anything we didn't talk about that you think is important to share, or anything you'd like to add?

Interviewee: No. I hope this had value. I thought your questions were well placed and good.

[Redacted to protect participant anonymity]

[End of interview]

Interview D

[Redacted to protect participant anonymity]

Interviewer: Okay, so now we can dive into the actual interview questions. One of the major objectives of the study is to describe the characteristics of a successful military interprofessional health care team, so to that end, I'm going to be asking you about what a successful team means to you. So my first question, it's kind of broad, but what are the characteristics of a successful military interprofessional health care team?

Interviewee: I think first and foremost is good communication between the team members and also a mutual respect for the viewpoint of those team members.

Interviewer: Interesting, okay. Any other characteristics you can think of between teamwork dynamic, interpersonal relationships, et cetera?

Interviewee: Not that I can think of right off, no.

Interviewer: Okay, that's fine, so do you think that in terms of good communication and mutual respect that you've listed as being characteristics of success, when you think about the different levels of care, for example, your experience in *[deployment location]*, your experience in forward surgical teams in *[deployment location]*, versus your experience…at *[hospital name]*, do you think that characteristics of successful teams have changed and can you think of any additional ones based off level of care, or do you feel like it tends to be pretty much the same across the board?

Interviewee: I personally think it's about the same across the board, however in those situations where the op tempo was a lot higher, such as it was in *[deployment location]*, then there has to be a higher level of trust, I think, in the team members and their abilities to perform as a member of the team in a professional manner, so as the team leader would not have to micromanage the care that's being given because of a higher level of trust and communication, quite honestly.

Interviewer: So what do you think establishes that level of trust?

Interviewee: I think that we do a very good job in the military… is when we send teams overseas, surgical teams specifically, we send them, even if they're 100 percent PROFIS, they come together for that period of pre-deployment usually at least two weeks, we're

utilizing ATTC down in *[location]* to build the teambuilding skills. It's a number of different things. Not only are we sending them all down there for surgical skills and trauma skills, but a lot of it is team building, and I think that's where you learn the team dynamics.

Interviewer: There's a word that you used I've never heard before. You said PROFIS?

Interviewee: Oh, PROFIS. It's professional filler system, so instead of the members being organic to that team and having trained over a period of a year at the same duty station, they will take professional surgeons, anesthesia, some nurses, and they may bring them from different areas…

Interviewer: So it sounds to me that you're saying that rather than 'cause I know there are some teams that work together, like you said, for a year prior to deployment. It sounds like under your system, those two weeks prior to deployment are part of that professional filler system that you're talking about. Am I on the right track?

Interviewee: No, not necessarily. So even a team that has been working together continuously in an organic unit, they may be 100 percent assigned to that team and they still will go down to *[location]* to the *[name of training center]* and train for two weeks.

Interviewer: I see.

Interviewee: Because, a lot of times, the surgeons, and the surgical techs, and the OR nurse, and the ER nurse, even though they're all assigned to that team, they may not have the opportunity to work together as a team.

Interviewer: I see what you're saying. Okay, thanks for clarifying that.

I'd like to ask you to think back across the care teams that you've either participated in as a health care professional or the teams you've overseen as a leader. Can you tell me a story of an event when a military interprofessional health care team proved themselves to be particularly successful?

Interviewee: Yes. When we were in *[deployment location]*, we were seeing on average *[number]* causalities per day for the first *[number]* months, sometimes more, and there was one particular incident, numerous incidents, but where because our dynamics were such that we were communicating very well together, we knew our

roles, the traumas were going much more smooth because everyone knew their jobs and everyone communicated their roles very well, and for instance, blood ration. If patients were coming in with multiple amputations, tourniquets may not have been applied very well and patients were exsanguinating. Our medics, our nurses, knew right off the bat that blood had to be warmed as quickly as possible, fresh frozen plasma had to be thawed, and without even communicating these things, we were all thinking on the same sheet of music. This happened on a daily basis, so it's not one particular case, but it was the way the traumas were managed. It became almost, to use a quote, poetry in motion.

Interviewer: So what do you think built the team into such a dynamic that was so easy to facilitate? You're saying that they didn't have to verbally communicate. Everybody just knew each other's roles. What built the team into being able to flow in that way?

Interviewee: I think first and foremost, it was the leadership from above. So our team leader, her leadership style was such that it fostered good communication, good trust, and I think it was the respect level that the top of the leadership had for even at the very bottom. There was just a good respect and no one felt belittled, no one felt their job was not important.

Interviewer: What made everybody feel like their job was important?

Interviewee: After each trauma, or each day, we would do an after-action report on how the day went, and so we communicated with each other and obviously when people reflect on their performance, we always would feel that, "I didn't perform up to par. I didn't do this. I didn't do that," whereas those around didn't notice that. We all felt that it did flow very well. So we, as human beings, tend to be harder on ourselves than we need to be, but the communication and the feedback during our after actions was such that everything did go very well.

Interviewer: Okay, that's great. So next question, can you tell me what you think makes interprofessional health care teams that work in the military different from those that work in civilian settings?

Interviewee: I think in the military, we do have more respect for each other as opposed to in the civilian side. I had a break in service… so I can speak to that. There's a tendency for the physician to have more of a feeling that they have to be in charge, versus in the military, your more experienced person may be the emergency room nurse or the ICU nurse who has had multiple deployments and multiple

experience, versus the surgeon who is right out of their residency. So I think that there's a lot more respect for that person based on their experiences.

Interviewer: Can you expand more on that? What are your thoughts in terms of dynamics in military interprofessional team collaboration in terms of the fact that we're not only asking these teams to work across these health care professional hierarchical differences but also differences in rank, in terms of the fact like you just said, the nurse on the team might have much more experience than the higher ranking surgeon. How do you think that impacts the collaboration of the care team?

Interviewee: Well, I think it's exactly opposite. Most of the time, your more experienced nurse is going to be a higher rank than that inexperienced surgeon because that inexperienced surgeon is most likely going to be a captain who is awaiting to pin on major, versus a possible senior major lieutenant colonel who has multiple deployments and multiple experience. So I think the rank structure does contribute to more of a respect, and you see that not only in a deployed setting but also in a large medical center or treatment facility in the states, but it does belong with that experience. When I am dealing with *[specialty]* residents or *[specialty]* residents… at *[hospital name]*, they see that I'm a *[rank]* and I have a little gray hair. I think that there's a level of experience that comes with that.

Interviewer: Okay, that makes sense. So next we'd like to understand if the kind of health care work that military interprofessional health care teams deliver is different than the work that interprofessional teams do in civilian context. So is there any difference in the kinds of work that we're asking these military teams to do that we wouldn't expect in a civilian team?

Interviewee: I don't think so, no. I think it's very similar. It's just a different dynamic, but I think it's very similar. I don't see the work that I did during my civilian years, the dynamics were a little different in then it was a fee for schedule, a fee for service, where everything depended on the dollar, but I think the dynamics were, or the interpersonal relationships were the same.

Interviewer: Do you feel like that's the same when you compare it from all your work in the austere environment as well?

Interviewee: Yeah. Obviously I think working in a high tempo deployed setting is much different than here and also is much different obviously than the civilian world. Number 1, you're asked to stay up 24

hours, 36 hours, doing the same thing over and over again, and it is taxing to you. That's something that doesn't happen even in our military MTFs and definitely not in civilian. Obviously you have other teams brought in. I think that there, again, the experience that the team goes through together as a whole is life changing.

Interviewer: Interesting, okay. So I'd like to ask you to describe the different health professionals that work in military interprofessional care teams. So we understand that some kinds of health professionals are unique to the military context. That is, there are some health care professional roles that are part of military teams but don't exist in civilian teams. Can you describe those unique military health professionals?

Interviewee: So on the team that I had in *[deployment location]*, we had surgeons, we had anesthesia, we had ICU nurses, ER nurse, OR nurse, and a physical therapist, and an ER physician. In *[deployment location]*, it was strictly surgical, so no physical therapy because it was resuscitative surgery. The patients didn't stay more than maybe 15, 20 minutes after definitive surgical care, so that was totally unique.

Interviewer: Okay, so now that we've had a chance to discuss the differences between civilian and military health care teams, I'd like to discuss the characteristics of what makes military care teams unique and then reflect on why those differences exist. So during our conversation so far, I've noted that certain characteristics that you've brought up in terms of what makes teams successful. So I've noted especially you've really reiterated communication and respect, good communication between team members, mutual respect for the viewpoint of those team members, having a high level of trust between team members, especially in the austere environment, and that being done because really from good leadership.

The team leader doesn't have to micromanage the care being given, trust has been established, also because of prior deployment experience, those two weeks leading up to the deployment that aren't just for your surgical and trauma skills but also for the teambuilding skills, and then that allows the team to know your roles, know your jobs, and be able to communicate their roles well, and with all that together, it gets everybody on the same sheet of music without really needing to communicate, right?

So you said that really builds up the team success, and then also the leadership from above in terms of the fact that if the leadership

fosters good communication and trust between team members, everyone has a feeling of respect, nobody feels belittled, every job feels important, and that's really facilitated also because of the after action reports where everyone has a chance to voice their thoughts and opinions on how things went, communicate with each other, and then continue to build that rapport between team members.

So is there anything in that list that I just reiterated for you that didn't sound right or anything you'd like to add in terms of what makes a military interprofessional health care team successful?

Interviewee: No, that was excellent, very good.

Interviewer: Okay, good, I'm glad. So with that list in mind I just reiterated for you, can you tell me why those characteristics are important for military interprofessional health care teams? So why are the characteristics that you've just told me are successful important for military health care teams?

Interviewee: Looking at it from a deployment setting, that's important because it does come down to a life-or-death situation sometimes for the team members themselves. That's totally different but might be expected. You would not see that in a civilian sector. Well, you just wouldn't, and the reason I bring that up is where we were in *[deployment location]*, we were very close to what's called the forward line of troops, the battle line, and so we would receive enemy fighters… as well, and so if your team wasn't functioning as a complete coordinated unit, some bad things could happen to the team while they're working on the casualty, being an explosive device that was on the patient. So I think that that in itself is just something totally different that you would not find not only in a civilian facility but also in a military facility anywhere in the country.

Interviewer: So one question. When you said [enemy] fighters, do you mean that you are providing care to some injured [enemy] fighters?

Interviewee: Correct.

Interviewer: Can you explain a bit of the dynamic of the team in terms of having to give that care?

Interviewee: We discussed that very early on before we even arrived in country because we knew the type of patients, where we were going, what was going to be expected of us, and was there going to be any problem? Did anybody have any problems taking care of potential

combatant? And that's something that the military drives home very hard before you ever go, that this is what we do. I had the same experience in *[deployment location]* dealing with [other enemy] fighters, too, so we discuss those things. We would do it as a group and it was just something that you're expected to do.

Interviewer: Do you think the fact that they were an enemy combatant ever affected in how the team would give care?

Interviewee: No.

Interviewer: That's great. I also heard you say about obviously you're in a war zone so you're out there on the battlefield. How do you feel like that affected the team just in terms of the danger of providing health care in the austere environment?

Interviewee: Well, there again, the leadership from above was so strong and in those situations, because the team is so small, you have to look after each other, and so we would always come together. No one was ever by themselves. Occasionally, they did, but we tried to do on our downtime things together, playing cards, watching a movie together, anything just to decompress and we would sit around and talk about the experiences that we had that day. We had a lot of casualties that didn't make it and those would affect you, and we had a lot of children that didn't make it, either, so those cases specifically needed to be discussed.

Interviewer: Interesting, okay. Can you tell me more about having that conversation? 'Cause it sounds like you guys did it over cards or a movie. What facilitated being able to have those conversations?

Interviewee: So we had a lot of young medics. I think our youngest was *[number]* years old, and so those of us who were more experienced and older, we knew that we needed to come together and try to decompress those issues with them, and it did affect some of them but we would make sure that we talked about them and get them to express their feelings. It was very informal. It wasn’t a formal sit down. It was maybe sitting outside… and talking about it…

Interviewer: … So it sounds like that really came together based on the experience of the older team members rather than a set instruction to do this for the younger –

Interviewee: Absolutely.

Interviewer: Wow, that's great. So again thinking back on all of your experiences, can you explain to me why you think the US military needs interprofessional health care teams that are specifically trained to work in the US military and what do you think the military should be doing to prepare its health professionals to be working in these interprofessional health care teams?

Interviewee: Hang on just a moment.

Interviewer: Yeah, no problem.

You're fine, no worries. Do you want me to repeat the question?

Interviewee: Yeah, please, if you don't mind.

Interviewer: Yeah, I figured. So what do you think the military should be doing to prepare its health professionals to be working in these military interprofessional health care teams? And actually right before that, I said can you explain to me why the US military even needs teams that are specifically trained to work in the military in the first place?

Interviewee: Well, I think the interprofessional teams that we have in the medical treatment facility in itself, or even *[hospital name]*, or overseas, those teams are very, very similar to what you find in a civilian facility. I think where we veer to more specialized teams is when it comes to fighting our wars and going into those zones. That's when those teams need to be more cohesive and different. So the care that we provide here at *[hospital name]* is identical to the care that is provided at *[civilian hospital name]*, or *[civilian hospital name]* in *[location]*, or whatever. It's, the care is identical. The teams, I'm willing to bet, are very close. The only difference is there's a rank structure in the military and so the level of respect is more, but I think where we see a difference is those teams that are sent forward for six months to a year living with a small team, working with a small team, MREs and using latrines, and sleep deprivation, that's where the preparation for those teams is important.

Interviewer: So what do you think that we should be doing to better prepare our health professionals to be working in these teams that you say are different because they have to deploy?

Interviewee: I think that the preparation for the teams themselves is actually quite good right now and I say that because over the past 16, 17 years, we have perfected them. When we first started sending

forward surgical teams over to *[deployment locations]* at the beginning of the wars, they were completely untrained, had never worked together as a team, and so were dysfunctional, until they, it took them… there was a learning curve for them. But since then, we have identified and there's a book, *Army Medicine Lessons Learned* from the deployments from the surgical teams, and what have we learned how to better prepare those teams, which they came up with the *[name of training center]* down at the *[location]* and have since started – I'm not sure how long ago they started that but I know I went through in 2010, so it was definitely before that, and so that's one method.

We've also started doing pre-deployment preparation with teams down at *[location]*, but on a unit level for those professional filler system professionals, there's nothing done here at *[hospital name]* for me to prepare to deploy with a team out *[location]*. Does that make sense?

Interviewer: Yes, it does. Okay.

Interviewee: But there's no formal training here to prepare me.

Interviewer: You have to be sent to those specific training facilities.

Interviewee: Correct. Once we identified that an individual is going to be deploying in the next six months, and usually it's about that period of time, once they are identified as deploying, then the train up starts. I think what would be better served, if you have a team that is say, for instance, a forward surgical team that is assigned to *[location]* and they're going to be pulling in surgeons, and anesthesia, and nurses from say four different MGFs throughout the country. At some point, I think that they would be better served during that four-month startup to bring everybody together for a week exercise and a get together at that home station, and then everybody can go back to finish what they're doing and meet up again for the final weeks. That, I think, would give a better cohesiveness to the team.

Interviewer: So that leads into my next question. I'd like to ask you about the ideal structure you'd envision for a military interprofessional care team, so if grass was always green, money never an issue, et cetera, how would you structure a military interprofessional care team to achieve maximum success?

Interviewee: To achieve maximum success, I think if they were all organic to that team to start with. So for instance, the forward surgical team

that I went to *[deployment location]* with was 100 percent organic from *[location]*. We all knew each other. We all worked together. That's not always fiscally capable of doing that, so in a great world, that would be the perfect scenario and it was for *[location]* because we were all there.

Interviewer: Can you explain what you mean by organic, specifically?

Interviewee: Organic meaning that everybody was assigned to that unit, so the forward surgical team we had in *[location]*, everyone was actually assigned. Their orders assigned them to that forward surgical team, and so on a weekly basis, the whole team got together for one day and did some type of a training exercise. Then the rest of the week, the surgeons, and the anesthesia, and the nurses would work at *[hospital name]*.

Interviewer: Yeah, that makes sense. So we've spent a lot of time talking about success, right, but sometimes to understand success, you have to understand failures. Can you give me an example of a military interprofessional health care team collaboration that did not work well and what characteristics of the team do you think contributed to this problem?

Interviewee: Yes, as a matter of fact, I can.

Interviewer: Unfortunately.

Interviewee: So I think the failure of a team specifically to the dynamics of a team boils down to poor leadership. When the leadership is caustic or dysfunctional, it leads to chaos amongst the whole team members, I think, and we had that situation in *[deployment location]*. The command of the team was dysfunctional and we knew that he was dysfunctional and caustic before we even left for *[deployment location]*, which caused quite a few complaints to come across before we even left... Because of continued caustic leadership in the deployed setting, he was eventually relieved of command, which definitely improved things, but it leads to chaos amongst the team members. It leads to unhappiness and when people were not trusting each other, then their performance lacked.

Interviewer: Right, okay. So I'd like you to think back on all your experiences in health care. Can you tell me what makes a military *[healthcare profession]…* different from a civilian *[healthcare profession]…* and why do you think that difference is important?

Interviewee: So looking from the viewpoint of a *[healthcare profession]*, the level of respect and the level of independence that I have as a military *[healthcare profession]* is far greater than what you would see in a civilian hospital. Now on a caveat on that, unless that is a rural hospital in the United States, which 68 percent of all of our hospitals are considered rural, where the *[healthcare profession]* is looked upon as a valuable team member. So when I say that, that is usually in a facility that has no *[specialty]* on staff and the *[healthcare profession]* is the primary *[specialty]* provider and therefore is looked upon by not only the surgical staff but the intensive care staff, the emergency room staff, as being the subject matter expert for all things *[specialty]* and *[specialty]*. That is as close to what you will find in the military as possible.

So in the military, *[healthcare profession]* are specifically trained in the military graduate programs for *[specialty]* to think and operate totally independent of anybody and that is because when we are deployed, we usually are all by ourselves, no other *[specialty]* providers, and it's just two surgeons and one *[specialty]*.

Interviewer: Okay, so based on your experience and this interview that we've had so far, can you give me a final definition of what you think is a successful military interprofessional health care team?

Interviewee: I would define it as a cohesive professional team that is able to work independent of each other but also, that doesn't make any sense, dependent on each other.

Interviewer: Take your time.

Interviewee: It's hard to explain and it goes back to the communication I was talking about earlier where a team can function… it's like I know what you're thinking without even having to hear you say it. Does that make sense?

Interviewer: Yes. That makes perfect sense.

Interviewee: So if I trust you and trust your skills and you trust my skills and my abilities, it is poetry in motion, and it comes down, once again, to communication, trust, and respect. I trust… I respect your skills and your experience and therefore I trust you and we can communicate.

Interviewer: Great. So before we end the interview, I want to make sure you had a chance to share all your thoughts and opinions. Is there

something we didn't talk about that you think is important or is there anything that you'd like to add?

Interviewee: Not that I can think of.

[End of interview]

Interview E

[Redacted to protect participant anonymity]

Interviewer: ...So my first question set is trying to figure out what are the characteristics of a successful military interprofessional health care team. Because one of the major objectives of our study is to describe the characteristics of a successful military interprofessional health care team. And so, to that end, we'd like to ask you about what a successful team means to you. So my first question is: in your opinion, what are the characteristics of a successful military interprofessional health care team?

Interviewee: I think that a team that openly communicates with one another. A team that has established expectations of their roles is important. I think it's necessary for a team to understand what their mission is, what they're working towards.

Interviewer: So when you're saying "openly communicate," can you go more in-depth on what you mean by openly communicating?

Interviewee: I mean that they meet on a regular basis, that they share information freely with one another. Like what I know you know. They share their data, their findings, that they are talking through – especially if it's dealing with a patient, having conversations on what the assessment is telling them, what the treatment plan is, being sure that they all agree. Especially when you're working with different services – like I've dealt a lot with having to work with a medical provider, a doctor, a psychiatrist, a case manager, all that – that everybody understands what the treatment plan is for the patient. That there's a common understanding of what the end goal is.

Interviewer: Yeah. So when you say – you said "necessary to understand their mission and understanding the end goal." Can you tell me more in your opinion what you feel that the mission is or that the end goal is?

Interviewee: Okay. I think that if you're working – when you have these teams working at a big command like this, I think it's important that all the teams have an understanding that the overall command mission is to support war-fighter readiness and taking care of families. So that everything we do is to support that. Whether or not you're the housekeeper or the billing person or the coder, the provider, what we do here feeds into that. And everybody has to understand that.

Because when you're working in a smaller team, when you have a team that's working with a specific patient, I think it's very important for them to all understand what the expected outcome is for the patient and for them to all agree that they're working towards that same outcome.

If the physician is more focused on the person being able to walk and the psychiatrist is more focused on controlling PTSD symptoms, then sometimes their goals are conflicting one another and treatment plans. And I think it's important that they come to a common understanding of establishing goals for their patient so everybody's working towards a shared goal.

Interviewer: Okay. Back to the shared goal. So these – I've really heard communication, role understanding, and mission focus, right? Of those characteristics you find for successful teams, do you feel like that changes based off the level of care a team is in? For example, with your experience in theater versus your experience in Garrison, do you think that the definition of what makes a team successful adapts or changes? Or do you feel like these characteristics you've given me are the same no matter what level of care you're providing?

Interviewee: I think that the characteristics remain the same. I think that when you're forward deployed, oftentimes the mission is obvious. For some reason there's clarity that comes with being in the... the mission is very clear. And sometimes I think that makes it easier 'cause you're not distracted by everything else that goes on. I think that in theater also – I mean, communication is just so vital in theater because, again, there's just so much going on. You have to be in constant communication with one another. You have to be talking to each other.

Interviewer: Okay. That's great. So I'd like you to think back across the care teams that you've either been a participatory member for as a health care professional or the teams that you've overseen in your leadership positions. Can you tell me a story of an event when a military interprofessional health care team proved themselves to be particularly successful?

Take your time.

Interviewee: So when I was in *[deployment location]* the second time, we were – we took incoming. So we had incoming come into the camp. So we had a lot of unexpected injuries because people were just wandering around the camp. And there was a surgical team located

on the camp. But where we were hit was probably a quarter mile from where the actual surgical team was. But in that case, it was the quick response. There were essentially corpsmen or medics on scene who were able to quickly respond, stabilize the patients, get transportation, quickly get them over to the surgical team, do turnover with the nursing staff of the patients' status and all that, and who then turned over to the surgeons as they're working through stabilizing the patients.

And then in the meantime, you have all the administrative staff working blood products and higher-level evacuation, calling in aircraft to evacuate the patients to a higher level of care. And it happened so fast and it happened so smoothly because there is the open communication. And these people with these life-threatening injuries are treated, transported, and stabilized within minutes because of the quick reaction and open communication with one another. And it's across multiple units. So it's not like sometimes they knew each other. They didn't know each other. So you're asking lots… instances.

Interviewer: What do you think facilitated such open communication in order to make all of that successful?

Interviewee: Because I think, again, in those settings you know what your mission is. I mean, it's just clear. Your mission is to treat this patient as quickly as possibly with all the tools that you have at your disposal. And that's what you're focused on. And that's what you do. Because there's no distractors. It's not like in Garrison when you're worried about your quality measures and your productivity measures and all that stuff on top of your day-to-day mission, right? …all those external distractors. When you're in theater like that, you know what you're supposed to do. You know what your primary focus is.

Interviewer: Right. Okay. So that kinda leads well into my next question. Can you tell me what makes an interprofessional health care team that works in the military different from those that work civilian settings?

Interviewee: I think that in the military setting, although there's competition, I think that really it's a lot more supportive. You have more to gain by supporting one another to make the mission successful by spending time trying to undercut your competition because you're trying to bring in more dollars to the clinic than another clinic. So there's more cooperation. I think that there's more willingness to

share resources, to share information. Because you do support a singular mission.

Interviewer: Okay. So next we'd like to understand if the kind of health care work that military interprofessional health care teams deliver is different from the work that interprofessional teams do in civilian contexts. So is there any difference in the kinds of work, in your opinion, that we're asking military teams to do that we wouldn't expect of a civilian health care team to deliver?

Interviewee: So are you talking about, I'm sorry, the care or just in general we expect them to do things that the civilian wouldn't have to do?

Interviewer: Both actually. …if you feel like there's things in terms of health care providers have to give on the clinical level that they wouldn't otherwise have to give, or just even outside of the clinical realm, do we ask –?

Interviewee: Yeah. I think on the clinical level, we're asking health care providers to not just treat the patient; we're asking the clinical team to assess the feasibility for continued service. We're asking the clinical team to: "In your best guess, do you think that this person is going to be healed enough to return to their work on a ship or their work with the Marines or their work here?" And we put a lot of pressure on them to make that decision. Because commanding officers of these units are counting on their people. And if they're not gonna be ready then they have to make decisions. I don't think we put that kind of pressure on our civilian counterparts to say, "Hey, do you think that this guy is going to be able to – decide now if in a year from now this guy's gonna be able to go back to welding" or teaching or whatever.

Interviewer: Right.

Interviewee: I think that we rarely make them make those kinds of decisions in the same that we do our military providers. I also think that, in a military setting, we have all the other external responsibilities. You're not just a provider. You're not just an administrator. You're not just a medical assistant. You're also a sailor, a soldier, an airman. And you have all your military responsibilities on top of that and what those responsibilities entail.

Interviewer: Do you think any of that has an impact on how a care team collaborates and interacts with one another? You know, whether it's in a positive or negative way. But the fact that every member of the care team is not only their health care professional role, but you

just said the multiple hats that you have to wear. On top of being asked these extra things that are asked of you in the military. Do you think that impacts teamwork collaboration, either positively or negatively?

Interviewee: I think it does. Because, again, I think that it can help it. Because you're all kinda sharing the same pain, which for some reason helps to build teamwork.

Interviewer: Yeah. Okay.

Interviewee: But also I think that the other thing is that I think that the military structure, the way that you have a rank and rate in the military, helps delineate authority clearer than maybe in a civilian facility.

Interviewer: Yeah. If you can expand more on that. Because we're really thinking about how you have professional hierarchy, which you find in the civilian context, in terms of your health care profession. And then there's also the rank structure. And sometimes they don't always align. You might have somebody like a nurse who outranks a physician. Can you describe how you feel like that impacts the collaboration of the care team?

Interviewee: Well, I think that it kind of – in some ways it helps delineate where certain responsibilities lie in certain settings. I think that we do a really good job, at least here in this facility – I think that we do a really good job about encouraging our people regardless of rank or position to speak up when they see issues with patient care or they're concerned about safety. But I think that having a delineated hierarchy also helps more junior personnel to know: "I can go to this person who outranks me and I can present a problem or an issue to them and they're gonna have the experience and maybe the power to do something about it." But in a civilian sector, it might be a little bit less clear as to who they could go to.

I also think that a lotta times with the way that our teams are set up in the military, when you're working with the junior enlisted and then you have nurses and physicians, they're really invested in each other's success, each other's professional success. And they do a lot to cross-train and train one another to enhance their skills and really push one another as a team. Because they want to see the people that they work with achieve professional success. So I'm not sure in a civilian sector if you have the same kind of dedication to one another.

Interviewer: Yeah. Why do you think that is? That you feel that there's this theme of dedication towards one another, towards wanting to see each other succeed. Why do you feel like you see that in these military interprofessional health care teams more so than in the civilian ones?

Interviewee: Because I think that, one, we spend a lotta time together. But oftentimes these are the same people that we are gonna deploy with, that we are gonna be in a combat zone with or on a ship with. And you have to trust them that not only are they gonna take care the patients but they're gonna take care of you too if something goes wrong. And you have to have that certain level of trust. I mean, that's why we say it: it's a different family. You really do become family. Because you're not just working with one another. And oftentimes the way that the military structure is, you often come back and work with these same people later on in your career too. So you just have that connection to one another. It really does become about essentially like a surrogate family.

Interviewer: How do you feel like you build that trust? Do you feel like it happens right when a team's put together that it's there? Do you feel like it has to be built over time?

Interviewee: I think it's a little bit of both. I think that having – when I see somebody with a certain level of rank, they automatically have a certain level of credibility with me because, to me, they wouldn't've gotten promoted to that level if they didn't have the experience, if they didn't have the time to achieve that. So they've already gotten a little bit of credibility.

Interviewer: Okay.

Interviewee: … I think helps build that right away. Of course when you meet new people, you're always gonna have to show me – show me what you got. But I think that we give them a little bit more room and a little bit more trust right off the bat because we know that they've been promoted so they must be doing something right.

Interviewer: Okay. So I'd like to ask you to describe the different health professionals that work in military interprofessional care teams. We understand that there are some kinds of health professionals who are unique in the military context. That is, there are some health care professional roles that are part of military teams but not

civilian teams. Can you describe those unique military health professionals?

Interviewee: Well, I mean, I guess we have corpsmen. I guess that's kind of unique. It's kinda like an MA in a civilian sector.

Interviewer: … Can you expand more on your perspective, in terms of the fact that we have these roles in the military and not in the civilian sector?

Interviewee: Well, because the corpsmen are specifically trained not only to work in a clinical setting but… also trained to work in a field setting. And… also trained to do a lot more things independently than you would allow a medical assistant to do without supervision. Just because of the nature of what [they] do. I mean, the corpsmen can carry medications on them. They could administer medications. They'll do things like, in emergency situations, do cricothyrotomies, chest tubes, needle thoracentesis, things like that that you wouldn't necessarily expect a medical assistant to do.

Interviewer: Do you feel like – what's your opinion on the level also of what's being asked of corpsmen to do? 'Cause it sounds like what you're saying are things that obviously would happen at a large modern hospital. Do you feel like there's also a level of what's asked of corpsmen to do medically in theater versus when they're in Garrison?

Interviewee: Oh, absolutely. When they're in theater, they're by themselves with 50 to 75 marines without any kind of medical provider for hundreds of miles. They are on their own. And I think that's the other thing a lotta the providers when they're in Garrison – a lot of our teams recognize that. So they put a lotta effort in training and preparing the corpsmen for those instances when they are gonna be out there all by themselves.

Interviewer: Right. Okay. So now that we've had a chance to discuss the differences between civilian and military health care teams and we've discussed the characteristics of what makes military care teams unique, I'd like to now ask you to reflect on why those differences exist. So during the conversation so far, I've taken some notes about what I've really heard you reiterate as what makes a military interprofessional health care team successful. So I've heard you first initially really mention open communication. And when you said "open communication," you mean meeting on

a regular basis, sharing information freely, whether that's data findings, information on the patient.

You mentioned that the team has established expectations of each member's role and that it's necessary to understand what their mission is, what the end goal is. And that could be – it felt like you said it was both the overall command mission – to support the war fighter, readiness, take care of families, etcetera. Everything goes into supporting that mission, whether it's the housekeeper or the provider. But also agreeing on the expected outcome for the patient. And you mentioned, for example: a physician might be focusing on the walking whereas a psychiatrist is focusing on the PTSD and their goals are conflicting. So mission focus in that aspect meaning that every team member needs to come to an understanding and have a shared goal with the specific patient as well.

I heard you mention that the characteristics remain the same despite the level of care that you're giving health care. Although in the forward-deployed environment, the mission feels really obvious and it lends some clarity to the sense of mission and there's less distraction and you feel like communication is really vital when you're in theater because there's so much extra going on.

And then finally you mentioned trust, how in the military that there's a sense of trust that is there and how rank actually kinda helps you establish a bit of trust when you know that somebody likely has experience because of their time that they've dedicated to the military and have been in that profession.

So, first of all, with that summary I just gave you, did that sound accurate? Is there anything that either sounded inaccurate or anything that you wanted to add?

Interviewee: No. That sounds good.

Interviewer: That sounds good? Okay. So with that list in mind, can you tell me why those characteristics are important for military interprofessional health care teams? So why are the characteristics that you've listed to me today as successful important for our military interprofessional health care teams?

Interviewee: Because I think that the communication – there's so much – I understand charting and I've read lots of charts and patient notes and stuff like that. But there's only so much you can put in those

things. And you can't – they're so one-dimensional sometimes. And when a care team comes together and actually discusses a patient and all the patient's idiosyncrasies, I think they get better insight into the patient. They also can see if there's conflicts in how they're assessing the patient, if one person is seeing something that the other person doesn't see. And then you have the case managers and the admin staff, the insurance guys, the case managers – those guys can oftentimes tell you, "Hey, this is a great care plan but it's not feasible because where this person is gonna go on convalescent leave is in the middle of nowhere and they're not gonna have access to this stuff so you need to come up with a different care plan."

You know, I think that those conversations – they can only happen when you're actually talking. You can't find that stuff just reading notes and passing notes back and forth to the providers.

Interviewer: Right.

Interviewee: I think that you also – again, as a team, you can talk about the overall mission. And when you're dealing with war fighters, the overall mission is to return the person to their unit. And, again, that goes back to them saying: "Ultimately the goal is to return this person back to their unit. But what is the feasibility of that? Is that really gonna happen? Or what time frame are we looking at? Rehab for what you're treating them for is three months but rehab for what I'm treating them for is six months. What's the likelihood of this person getting back up on their feet permanently?"

And, again, all that communication feeds into the overall mission. And I think in the civilian sector, it's all about productivity and revenue, right?

Interviewer: Yup.

Interviewee: And although we are concerned about productivity at our level, really our revenue is generated in how many of these people we get healthy and back out to doing their jobs. So it's just I think a different focus and that's why communication is so critical to us.

Interviewer: Right. Okay. So, again thinking back on all your experiences, can you explain to me why the US military needs interprofessional health care teams that're specifically trained to work in the military? And then what do you think that we should be doing to prepare our health professionals to work in these military interprofessional health care teams?

Interviewee: I think in the military, you have to understand what the military is. What makes us unique. When you bring in providers – and sometimes we see it with the brand-new providers who are joining the team, the interns or whatever, who have had very little military experience. Here in the military, you have to understand what your second- and third-order effects are. Right?

If you have a rifleman who is injured and has a blast injury, and maybe it's not bad – maybe they only just need a few days off the line to recover – you can't just dope them up and send them on their way like maybe you would in a civilian sector. No. You have to ask yourself, "Okay, well, I could medivac this person back to the States." But then you have to understand in the military setting: what does that mean? Well, that means that I have 1 more rifleman missing from a squad of 12, and 1 person means a lot when it's only 12 people that you're talking about.

And you have to understand that it takes at least 100 days to get a replacement for that person back into theater. And that means that there's one less person on the battlefield doing what they're supposed to be. And if this is a unit of 500 and they've had 100-plus injuries, you're rendering the unit battle ineffective, which means that they're not meeting their mission, which means that they're not taking control of a city that they should be taking control of. You have to understand all that. You really do.

Interviewer: So what do you think the military should be doing then to better prepare our teams to be working in these military-specific interprofessional teams?

Interviewee: I think that a lotta times a lot of our team members – like I've been very fortunate and have moved around in my career. I have professionals that have been here for eight, ten years, this clinical setting. And maybe they're less familiar with how it really means to be operational and how the decisions that they make affect operations and stuff like that. So I do think that we need to do a better job of training our military professionals on probably strategy and tactics and how the decisions they make can affect those decisions being made at the tactical level, and sometimes even at the strategic level.

Interviewer: Okay.

Interviewee: The military operations.

Interviewer: Okay. Great. Next question. I'd like to ask you about the ideal structure that you'd envision for a military interprofessional care team. So if the grass was always green, money never an issue, how would you –

Interviewee: I missed the first half of what you said. It kinda faded out a little bit.

Interviewer: That's okay. I'd like to ask you about the ideal structure that you'd envision for a military interprofessional care team. So, in your world and in the work that you've been doing, if grass was always green, money wasn't an issue, etcetera, how would you structure a military interprofessional care team to achieve maximum success?

Interviewee: That's a tough one.

Interviewer: Yeah.

Interviewee: Well, it's tough because, again, it's based off of: each individual unit has a mission. But I think ideally, medical teams would have – you would have some kind of case manager administrator to manage follow-on care for patients, especially patients that are evacuated. You'd have a doctor. You'd have a nurse. You'd probably have a couple of corpsmen. You'd have a social worker to deal with mental health issues and things like that. And then did I say an administrator?

Interviewer: Yes. Okay. So it sounds like you feel like you'd really include a lot of roles on the team. Am I interpreting that correctly? …you'd bring in a lotta different –

Interviewee: I would. I would have the overarching teams and then you'd have probably – at the lowest level, I think you would have the corpsman and a nurse or the corpsman and the doctor working together. I think the overarching team and then you kind of mix and match it as needed I guess.

Interviewer: So we've spent the past 40 minutes or so talking about success. I wanted to ask you a question though on failure. So can you give me an example of a military interprofessional health care team collaboration that did not work well? And what characteristics of the team do you think contributed to this problem?

Interviewee: I mean, I've seen failure when – the biggest failure that I've seen is when *[position]* makes wide-sweeping decisions and forcefully

implements them without really understanding what's going on on their lower levels. *[Redacted to protect participant anonymity]*.

Interviewer: Why do you think it happens? What do you think are the characteristics that contribute to the issue?

Interviewee: I think it's not communicating. It's not validating the information that you have. I think that sometimes they make decisions based off of very few data points that really haven't been validated or aren't representative of the wide variety of settings that we have. And I think that sometimes they don't pre-brief what their plan is ahead of time to allow people to provide feedback to.

Interviewer: Okay. So I have two more questions for you. I'm trying to remain cognizant of your time. And we're approaching ten minutes until it's been an hour. So my next question for you is: I'd like you to think back on all your experiences in health care; can you tell me what you feel makes – you have your experience as a *[healthcare provider]*... Whatever the equivalent you feel like exists in the civilian sector, how do you feel like doing those roles in the military environment – what makes that job different in the military versus doing that job in the civilian sector? And why do you feel like that difference is important?

Interviewee: So, for me – I think that's a tricky question.

Interviewer: It is.

Interviewee: Because in the military, I have to be invested in my person, and their conduct off duty is just as important as their conduct on duty. You know what I mean? So I'm dealing with all the operations management, personnel management, making sure I have certain positions filled and all that. That's one aspect of it. But then when somebody gets a *[infraction]* or somebody gets in trouble for *[infraction]*, I have to deal with all of that too.

Interviewer: Right.

Interviewee: My civilian counterpart would say, "Yeah, I really don't care if you got a *[infraction]*. You need to go to court, take time off, and do what you gotta do." Right?

Interviewer: Right.

Interviewee: "You've got a *[infraction]* charge against you? Well, come see me when the court's made its ruling and then we'll go from there. But I

don't care other than that." And I have to be focused on the professional development of my subordinates too. Because I have to train them to move up in the organization. And I think that sometimes in the civilian sector, although I do think that some are doing a better job of trying to grow their own, it used to be: "Rather than grow my own, I'll just hire somebody who's already trained up to fill this new position." For us, we're building a legacy. So we're committed to developing our subordinates because we truly expect them to grow into roles of greater responsibility within the organization.

Interviewer: Right. Okay. So, based on this interview, based off your experiences – this is my last question for you – can you give me a final definition for a successful military interprofessional health care team?…

Interviewee: ...I think a successful team is committed to open dialogue, sharing of information and ideas, has a shared understanding of the mission and the desired end state, and are committed to one another's success and the command's success.

Interviewer: Okay. Great. So, before we end the interview, I wanna make sure you had a chance to share all your thoughts and opinions. Is there something we didn't talk about that you think's important? And is there anything you'd like to add?

Interviewee: Nope. I think that's all I got.

[End of interview]
